# Human β defensin type 2 based novel antimicrobial peptide prediction using both molecular dynamics simulation and artificial intelligence methods

**DOI:** 10.3389/fchem.2026.1913090

**Published:** 2026-09-29

**Authors:** Ishrat Jahan, Hailemicael Lulseged Yimer, Kushal Badal, Michaela Brady, Jason J. Guo, Bryce Richardson, Letu Qingge, Liqun Zhang

**Affiliations:** 1 Chemical Engineering Department, University of Rhode Island, Kingston, RI, United States; 2 Department of Computer Science, North Carolina Agricultural and Technical State University, Greensboro, NC, United States; 3 Department of Chemistry and Chemical Biology, Barnett Institute for Chemical and Biological Analysis, Northeastern University, Boston, MA, United States; 4 Chemical Engineering Department, Hampton University, Hampton, VA, United States

**Keywords:** AI modeling, binding, human beta defensin 2, molecular dynamics simulations, RBD, SARS-CoV-2

## Abstract

The global health crisis triggered by SARS-CoV-2 has highlighted the urgent need for effective treatments. Although several anti-viral drugs targeting SARS-CoV-2 have been developed, there remains considerable interest in exploring natural antimicrobial peptides (AMPs) secreted by the human innate immune system as alternative therapeutic agents. Human β defensin type 2 (hBD-2) has been found to bind to the receptor-binding domain (RBD) of SARS-CoV-2 and block the virus entry. In order to design effective anti-virial drugs based on hBD-2, 32 block-mutants (mutation at more than one site simultaneously) were designed, and their interaction with RBD were predicted in terms of structural stability and binding energy. In combination with data from a total of 247 hBD-2 point-mutants reported in our previous work, artificial intelligence (AI) models were trained to design more efficient hBD-2 variants. With that, advanced AI approaches, including convolutional neural network (CNN) and long short-term memory (LSTM) models were employed to predict hBD-2 mutant sequences with potentially enhanced binding affinity with RBD compared with the wildtype peptide. Based on the predicted sequences, the AlphaFold program was applied to predict the structures of AMPs, and then all-atom molecular dynamics (MD) simulations were performed to calculate the binding interaction energies between each hBD-2-based AMP and the RBD as a measure of binding affinity. Structural stability was also evaluated through analyses of Root Mean Square Deviation (RMSD), Root Mean Squared Fluctuation (RMSF), the number of hydrogen bonds formed, and buried surface area. The results indicate that several AMPs exhibited more stable binding with RBD than the wildtype, highlighting their potential as novel hBD-2-derived antiviral candidates against SARS-CoV-2. Furthermore, based on the obtained results, the LSTM model demonstrated greater effectiveness than the CNN model in designing novel hBD-2-based anti-virial drugs against CoV-2.

## 1 Introduction

Severe Acute Respiratory Syndrome Coronavirus 2 (SARS-CoV-2), which has caused more than 7 million deaths worldwide up to now, has emphasized the essential requirement for a thorough comprehension of viral-host interactions to facilitate the development of effective treatments (Khavani et al., 2023). Coronaviruses are RNA viruses that undergo continuous mutation and may develop increased transmissibility or enhanced resistance to immunity acquired from previous infection or vaccination (Zhang and Li, 2024). A crucial element of coronaviruses is the Spike (S) protein, which binds to host cell receptors and facilitates viral fusion with cell membranes (Siu et al., 2008), (Yoshimoto, 2020), (Walls et al., 2016). This binding process involves dynamic conformational changes within the receptor-binding domain (RBD) of the S protein (Wrapp et al., 2020), enabling interaction with the angiotensin-converting enzyme 2 (ACE2) receptor on host cells (Yan et al., 2020), (Lan et al., 2020), (McCallum et al., 2020).

In order to inhibit virus infection, various strategies have been employed to develop antiviral drugs and therapeutics agents (Mei and Tan, 2021), (Zhang et al., 2020), (Riva et al., 2020) (Fleming, 2018), (Ju et al., 2020), (Russell et al., 2020), (Vankadari, 2020), one of which is to introduce antimicrobial peptides (AMPs) capable of blocking the interaction between RBD and ACE2. Humane β defensin type 2 (hBD-2), a natural AMP secreted from the human innate immune system, has been found to bind with RBD of SARS-CoV-2 tightly, thus can block the binding of RBD with ACE2 (Zhang et al., 2022). Another defensin, human α defensin 5 (HD5), has been found to bind with ACE2 receptor instead, thus also can block the binding of ACE2 with RBD (Wang et al., 2020). The cathelicidin LL-37 can play a key role in early defense against the SARS-CoV-2 (Ghosh and Weinberg, 2021). However, with the rapid evolution of SARS-CoV-2, hBD-2 has been found to be inefficient combating advanced variants of SARS-CoV-2 (Zhang and Li, 2024). Based on ΔΔG predictions from our previous study (Zhang and Li, 2024), hBD-2 is expected to exhibit a similar neutralization effect on the Alpha variant to the original SARS-CoV-2 virus, and the Beta variant should be able to escape the neutralization effect of hBD-2. In order to design novel hBD2-based drugs with stronger binding affinity with RBD than the hBD-2 wildtype, it is essential to investigate the interaction between hBD-2 mutants and RBD. Up to now, in total 247 spot-mutations at 13 sites (RES18-30) of hBD-2 have been generated and all-atom MD simulations have been conducted to predict the binding affinities of these hBD-2 mutants with RBD. The results indicated that only a subset of the mutants exhibited enhanced binding relative to hBD-2 wildtype (Jahan and Zhang, 2025). In order to design potentially more effective hBD-2-based anti-virial drugs and further explore the relationship between hBD-2 sequence and RBD-binding affinity, 32 block-mutants each has mutations at more than one site in RES18-RES30 region simultaneously, were designed and their interactions with RBD were predicted in this study. Then, by combining the results from the 247 spot-mutants and the 32 block-mutants, we investigated the application of combining MD and advanced AI modelling to predict novel hBD2-based antimicrobial peptides with enhanced binding affinity with SARS-CoV-2 RBD while maintaining sufficient structural stability.

Protein stability prediction in the context of mutations is an ongoing challenge within the fields of protein engineering and drug development. Traditional experimental approaches for determining changes in Gibbs free energy (ΔG) tend to be both time and resource intensive, prompting the need for more efficient computational techniques (Torrisi et al., 2020), (Narayan et al., 2017). Recent advancements in AI have offered promising avenues for addressing this complex issue (Yang et al., 2020). In this study, two deep learning models were employed to estimate ΔG changes in proteins mutated at 13 sites of hBD-2. First, a Convolutional Neural Network (CNN)-based approach was used to extract sequence-based features pertinent to protein stability, as CNNs have demonstrated strong capability in capturing local patterns within biological sequences (Öztürk et al., 2018). Second, a Long Short-Term Memory (LSTM) network was applied to capture long-range sequential dependencies within the peptide sequence, which are critical for understanding how distal mutations affect overall structural stability and binding affinity. Together, these complementary models provide a rapid framework for evaluating the effects of hBD-2 mutations on protein stability and identifying novel peptide candidates with enhanced binding affinity for the SARS-CoV-2 RBD.

## 2 Materials and Methods

### 2.1 247 spot-mutation simulation results and 32 block-mutation MD simulation results were applied to train AI model

In total 247 hBD-2 spot-mutations were built by mutating each residue from RES18 to RES30 of hBD-2 from the current residue to the other 19 residues (the sequence of hBD-2 is shown below), and their binding free energies with RBD were predicted in our previous work (Jahan and Zhang, 2025). It was found that 11 spot-mutants of hBD-2 exhibited binding affinities comparable to or greater than that of hBD-2 wildtype. After repeating the simulations for these 11 mutants, consistent results were obtained in terms of the binding affinity and structural stability (result shown in our recent publication (Jahan and Zhang, 2025)). These 247 mutant sequences and their corresponding structural stability and binding affinity data were used to train AI models to predict the optimal hBD-2 mutant sequences with enhanced binding affinity with RBD.

Besides that, in order to increase the size of the dataset used for AI model training, a series of block-mutations was introduced within the RES18-RES30 region of hBD-2 (in bold and underlined). These block-mutants were generated by simultaneously mutating more than one sites based on combinations of the 11 beneficial point-mutations identified in our previous study (Jahan and Zhang, 2025), using the original hBD-2 sequence as the template, as shown below.

GIGDPVTCLKSGAICHP **^18^VFCPRRYKQIGTC^30^** GLPGTKCCKKP.

In total 32 multiple-site hBD-2 mutants (block-mutants) were generated, and MD simulations on the hBD-2 block-mutants binding with RBD were set up and performed. For each of the 32 block-mutants, the initial structure of the hBD-2 mutant was first predicted using AlphaFold2. Subsequently, HADDOCK docking was employed to predict the initial stable binding structure of hBD-2 mutant and RBD, with the details shown in Supplementary Table S1 in SI. The MD simulation results for the 32 block-mutants are summarized in Supplementary Table S2, including the average RMSD, the binding interaction energy, and the number of hydrogen bonds formed during the simulations. Among the 32 mutant sequences, 7 (Seq6, Seq9, Seq11, Seq23, Seq27, Seq30 and Seq31) exhibited promising binding characteristics. Therefore, additional replicate simulations (reruns) were conducted for these seven candidates to validate the initial findings. The simulation results obtained for the 32 block-mutants were subsequently incorporated into the dataset used to train the AI models.

### 2.2 Data processing for AI modeling

#### 2.2.1 CNN model

CNN model details. We developed a robust data processing pipeline to prepare protein sequence data for predictive modelling of ΔG values following mutations on 13 residues of hBD-2 sequence. Using Python and the Pandas library, we transformed amino acid sequences into numerical representations and integrated time-series data. This process involved encoding each protein sequence was encoded as a set of numerical values and paired with the corresponding time points. The resulting dataset comprised feature vectors (X_train_) containing the encoded sequences and time data, alongside their associated experimentally determined ΔG values (y_train_). For instance, the sequence VFCPRRYKQIGTC at 0 ns with ΔG = −22.1 kcal/mol was transformed to X = [86, 70, 67, 80, 82, 82, 89, 75, 81, 73, 71, 84, 67, 0], y = −22.1. This approach preserved both sequence specific information and ΔG values, creating a comprehensive foundation for machine learning model development. We split the data into training (80%) and test (20%) sets, and applied StandardScaler for normalization. We employed StandardScaler from scikit-learn to normalize our input features and target values. This scaler standardizes features by removing the mean and scaling to unit variance. For each feature X, it computes:

$$X_{scaled}=\frac{X-\mu }{\sigma }$$ (1)

where µ is the mean and σ is the standard deviation of that feature across all samples. This transformation centers the data around 0 with a standard deviation of 1, which often improves the performance and convergence of machine learning models, particularly neural networks. We applied this scaling separately to our input features (X) and target values (y) to ensure consistent scaling across all variables in our model. Our preprocessing methodology effectively bridged the gap between raw experimental data and input required for sophisticated predictive algorithms, enabling the creation of models capable of predicting ΔG values for new protein sequences mutated with 13 residues of hBD-2 sequence. The CNN Model Hyperparameters applied in the modelling are shown in Supplementary Table S3.

The Proposed CNN Architecture in AI modelling. For predicting ΔG values, we employed a Convolutional Neural Network (CNN) using TensorFlow and Keras. The model architecture consisted of two Conv1D layers (32 and 64 filters, kernel size 3) with ReLU activation, followed by a Flatten layer and two Dense layers (64 units with ReLU and 1 unit with linear activation). The model was compiled with Adam optimizer (learning rate 0.001) and Mean Squared Error loss function. Training was performed for 100 epochs with a batch size of 32, using 20% of the training data for validation. Figure 1 shows our CNN model architecture.

**FIGURE 1:**
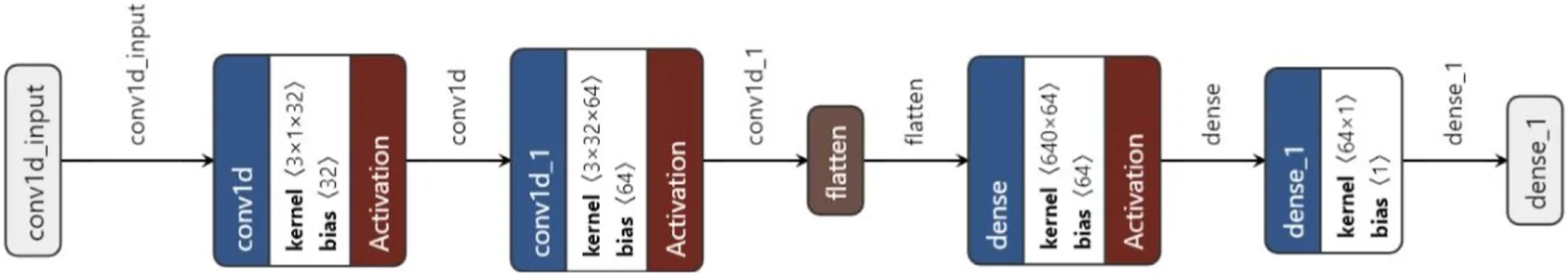
Diagram of the CNN architecture: a sequence and time input passes through two one-dimensional convolution layers with ReLU activation, followed by flattening, two dense layers, and a single continuous ΔG output.

Model performance was evaluated using Mean Absolute Error (MAE), Root Mean Squared Error (RMSE), and R^2^ score on both training and test sets. This approach allowed us to assess the model’s predictive capability for ΔG values of protein sequences mutated with 13 sites of hBD-2. The hyper-parameters of the CNN model are shown in Supplementary Table S3.

#### 2.2.2 LSTM model

LSTM model introduction. We further developed a robust data processing pipeline specifically designed for long short-term memory (LSTM)-based temporal protein analysis. Using Python with Pandas and PyTorch libraries, we implemented a novel encoding scheme where amino acid sequences were mapped to integer indices rather than one-hot vectors, preserving sequence information more efficiently. Each of the 20 standard amino acids was assigned a unique integer (1–20), with 0 reserved for padding and 21 for unknown residues. For instance, the sequence VFCPRRYKQIGTC was encoded as [18, 5, 2, 13, 15, 15, 20, 9, 14, 8, 6, 17, 2]. This integer encoding approach is particularly well-suited for LSTM architectures as it allows the embedding layer to learn meaningful representations for each amino acid during training. Unlike one-hot encoding which creates sparse high-dimensional vectors, our method produces compact representations that capture amino acid relationships through learned embeddings. The temporal dimension was integrated by pairing each encoded sequence with its corresponding simulation time point (401–500 ns). Each sample consisted of an encoded sequence tensor, a time value, and its associated ΔG measurement from MD simulations. We split the data into training (80%, 22,560 samples) and test (20%, 5,640 samples) sets. We employed StandardScaler from scikit-learn to normalize both time values and target ΔG values independently. This scaler standardizes features by removing the mean and scaling to unit variance.

For each feature X, it computes using Equation 1. This transformation centers the data around 0 with a standard deviation of 1, which often improves the performance and convergence of machine learning models, particularly neural networks. We applied this scaling separately to our input features (X) and target values (y) to ensure consistent scaling across all variables in our model. Our preprocessing methodology effectively bridged the gap between raw experimental data and input required for sophisticated predictive algorithms, enabling the creation of models capable of predicting ΔG values for new protein sequences mutated with 13 residues of hBD-2 sequence. The LSTM Model Hyperparameters applied in the modelling are shown in Supplementary Table S4.

The Proposed LSTM Architecture in AI modelling. For predicting ΔG values across simulation time, we employed a LSTM neural network using PyTorch. The model architecture incorporated three main components: (1) an Embedding layer that transformed integer-encoded amino acids into 10-dimensional dense vectors, enabling the network to learn meaningful representations of each amino acid; (2) a 2-layer LSTM with 64 hidden units processing the embedded sequences to capture sequential dependencies and temporal patterns; and (3) a regression head combining the LSTM output with the time input through fully connected layers (65→32→1 units) with ReLU activation and dropout (p = 0.2) for regularization. The LSTM architecture is particularly well-suited for protein sequences as it can capture long-range dependencies between residues through its gated memory cells. The model used packed padded sequences to efficiently handle variable-length inputs (12–13 residues). It was compiled with Adam optimizer (learning rate 0.001) and Mean Squared Error loss function. Training was performed for 100 epochs with a batch size of 32, using an 80/20 train-test split. The total model contained 55,101 trainable parameters. Figure 2 shows our LSTM model architecture.

**FIGURE 2:**
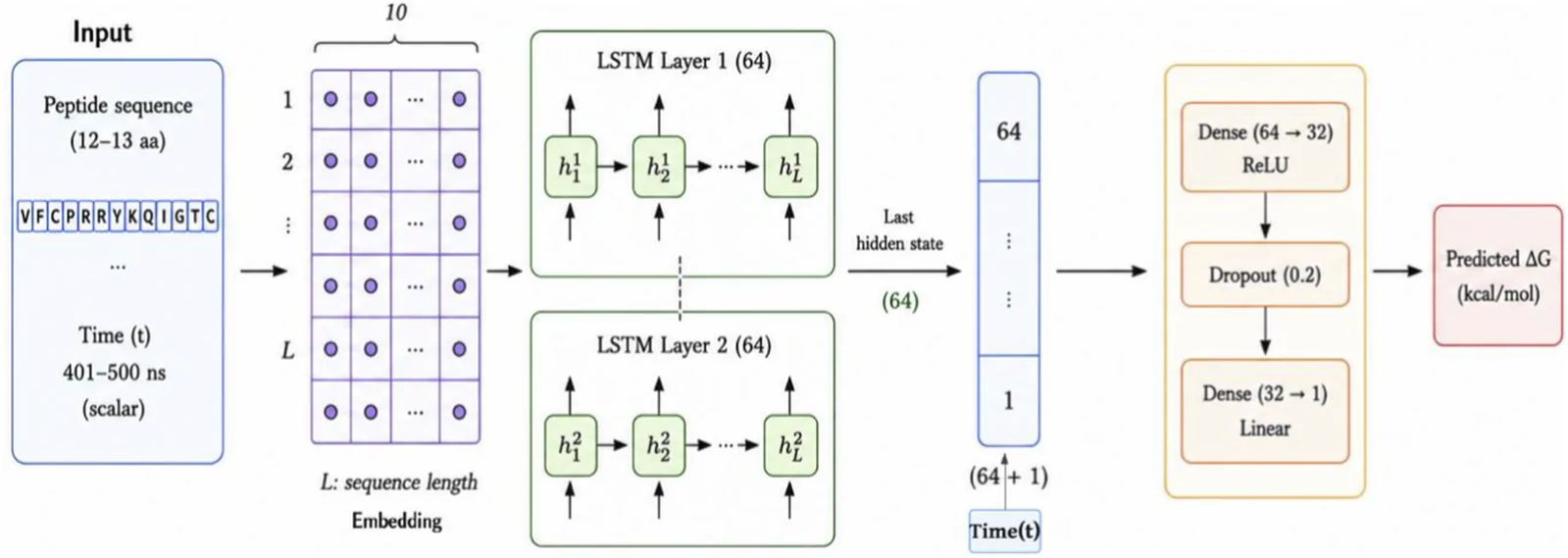
Diagram of the LSTM architecture: integer-encoded peptide sequences are converted to 10-dimensional embeddings and processed by two 64-unit LSTM layers. The sequence representation is combined with time and passed through dense, dropout, and linear layers to predict ΔG.

Hyperparameters were selected through iterative tuning, not fixed *a priori*. For the CNN, we tested different filter sizes and layer counts, settling on two Conv1D layers (32, 64 filters) based on lowest validation loss. For the LSTM, we tested embedding sizes, hidden units, and dropout rates (0.1–0.3), settling on a 10-dimensional embedding, 64 hidden units, and dropout p = 0.2 based on the smallest train-validation gap. Learning rate (0.001) and batch size (32) followed standard Adam defaults, confirmed by stable loss curve convergence for both models.

Model performance was evaluated using Mean Absolute Error (MAE), Root Mean Squared Error (RMSE), and R^2^ score on both training and test sets. This approach allowed us to assess the model’s predictive capability for ΔG values of protein sequences mutated with 13 sites of hBD-2 across simulation time. The hyper-parameters of the LSTM model are shown in Supplementary Table S4.

### 2.3 ML predicted hBD-2 mutant secondary structure prediction using AlphaFold2 colab

Based on simulation results obtained for 247 spot-mutants and 32 block-mutants, AI models were trained, and subsequently used to predict in total 50 novel multiple-site hBD-2 mutants, including 30 mutants generated using the CNN model and 20 mutants generated using the LSTM model. In order to run all-atom MD simulations on these 50 hBD-2 mutant sequences, AlphaFold2 Colab was applied to predict their secondary structures.

#### 2.3.1 hBD-2 mutant secondary structure prediction using AlphaFold2 colab

AlphaFold2, developed by DeepMind (Jumper et al., 2021), (Tunyasuvunakool et al., 2021) and its Colab implementation (Mirdita et al., 2022) have greatly facilitated accurate protein structure prediction. In this study, we predicted the three-dimensional structure of peptides using the AlphaFold2 Colab notebook based on the sequences of 32 manually designed hBD-2 block-mutants and 50 hBD-2 mutants generated through machine learning. The notebook is accessible at the following link: https://colab.research.google.com/github/sokrypton/ColabFold/blob/main/AlphaFold2.ipynb. The predicted sequences were submitted to the AlphaFold2 Colab notebook to generate structural modes. Subsequently, the predicted structures were analyzed using PyMOL (Schrödinger and DeLano, 2020) to evaluate the effects of different mutations on the structural stability and potential functionality of hBD-2. Structures with higher predicted Local Distance Difference Test (pLDDT) scores were prioritized, as higher pLDDT values indicate greater confidence in the predicted local structure. In addition, the predicted Template Modeling (pTM) score, which measures the overall accuracy of the global fold, was considered. Structures with pTM scores below 0.5 were considered to have low confidence in their global fold topology. The results for the 30 sequences generated from CNN model, presented here as representative examples, are summarized in Supplementary Table S2.

#### 2.3.2 Molecular docking details

The hBD-2 mutant structures obtained from the AlphaFold2 Colab notebook were used for molecular docking with the RBD. HADDOCK 2.4 (High Ambiguity Driven biomolecular Docking) (Honorato et al., 2024) was applied to simulate the interactions between the AlphaFold2-predicted hBD-2 structures and the RBD. Specifically, the RBD structure obtained from our previous work (Zhang et al., 2022) and the hBD-2 mutant structures predicted by AlphaFold2 were first pre-processed to ensure that they were correctly formatted and free of structural inconsistencies. Next, the binding sites were selected based on prior knowledge and experimental evidence (Zhang et al., 2022), (Zhang and Li, 2024) to guide the docking procedure. Finally, the resulting complexes were refined through explicit-solvated simulations and energy minimization. Supplementary Table S1 summarizes the molecular docking results and highlights the complex structures with the lowest HADDOCK scores.

### 2.4 Molecular dynamics simulations details

Gromacs simulations. The initial binding structures of hBD-2 mutant and RBD from both manually mutated and ML predicted, were applied to set up all-atom molecular dynamics simulations. We ran a total of 50 simulations for ML predicted sequences (30 from CNN model and 20 from LSTM model), and 32 simulations based on manual prediction, each lasting 500 ns, following the same protocols as described in the MD simulation section in our previous work (Jahan and Zhang, 2025) using GROMACS-2023.1 (Van Der Spoel et al., 2005) package with CHARMM36-jul2022 force filed (Huang et al., 2017). In brief, during the simulation set-up, disulphide bonds between the cysteine residues (Cys11-Cys40, Cys18-Cys33, Cys23-Cys41) were conserved whenever possible. The hBD-2&RBD complexes were solvated with the TIP3P water model in a cubic box under periodic boundary conditions, while maintaining the 12 Å minimum distances between the protein atoms and the box boundaries. The net charge of the system was neutralized by adding sufficient chloride ions. After that, systems were subjected to the steepest-descent minimization for 100 ps, followed by two staged equilibrations at standard thermo- and barostat (v-rescale-temp for NVT ensembles, 1 ns at 310 K, followed by the Parrinello-Rahman barostat for the NPT ensemble, 5 ns at 1 atm and 310 K). MD simulations were run for 500 ns each (total 41 µs) under NPT ensemble and Particle-Mesh-Ewald algorithms were used for computing long-range electrostatic interaction. For 32 block-mutants of hBD-2 binding with RBD, we used the hBD-2 wildtype bound with RBD as the initial structure to set up the all-atom simulations. The simulation set-up was the same as above for those 50 hBD-2 mutant binding with RBD simulations.

To assess the structural stability of the complexes, the average root mean square deviations (RMSD) of the backbone atoms were calculated using the gmx rmsdist tool from the GROMACS suite. Additionally, the GMX/MMPBSA program (Genheden and Ryde, 2015) was employed to calculate the interaction energy between hBD-2 sequences and RBD during the last 100 ns of the simulations. The number of hydrogen bonds formed between hBD-2 and RBD in both wildtype and mutant forms was determined using the gmx hbond command in GROMACS. A distance cut-off of 0.35 nm and an angle cut-off of 30° between the donor-acceptor-hydrogen atoms were applied in the hydrogen bonds calculation. Buried surface area (BSA) analysis was calculated for hBD-2&RBD complexes. The BSA of the complex was determined through a three-step process using the gmx sasa command from GROMACS (Eisenhaber et al., 1995).

NAMD simulations. For the promising hBD-2 mutants which can enhance the binding with RBD based on above Gromacs simulation predictions, repeat simulations using NAMD program (Phillips et al., 2005) were also conducted. Starting from the same initial structure, NAMD simulation was set up using VMD program (Humphrey et al., 1996). Each system was solvated with enough TIP3P water molecules to have the minimum distance from protein to the box edge to be at least 12 Å. After that, 0.15 NaCl solvent was added to neutralize the systems.

After a brief energy minimization using the conjugate gradient and line search algorithm, 4 ps of dynamics was run at 50 K, and then the system was brought up to 310 K over an equilibration period of 1 ns using NAMD program. All-atom molecular dynamics simulation was performed on each system using NAMD program and CHARMM36 forcefield (Huang et al., 2017). The temperature was 310 K and a desired pressure of 1 atm using standard thermo- and barostat. The sampling run was 500 ns with a time step of 1 fs? The trajectory output frequency was 1 ps?

To analyze the trajectories, the Root Mean Square Deviation (RMSD) and Fluctuations (RMSF) of the proteins were calculated using the VMD program and an in-house analysis script based on the coordinates of the backbone Ca atoms after aligning the trajectories respectively, to the original crystal structure of the RBD, the hBD-2 mutant (hBD-2m) predicted from AlphaFold, and to the initial complex structure of the RBD and hBD-2m predicted from HADDOCK docking. The buried surface area (BSA) for the complex was calculated in two steps using the VMD program and a script using the Richards and Lee method with the water probe size of 1.4 Å (Lee and Richards, 1971). First, the total solvent accessible surface area of the complex (ASA_complex_) was calculated based on the complex’s trajectory. Second, the accessible surface area of each protein in the complex (ASA_RBD_, ASA_hBD-2m_) was calculated for each protein individually. Then, the buried surface area (BSA) is calculated using Equation 2:

$$\text{BSA}=0.5*\left({\text{ASA}}_{\text{RBD}}+{\text{ASA}}_{\text{hBD}-2m}\;–\;{\text{ASA}}_{\text{complex}}\right)$$ (2)

The number of hydrogen bonds between the RBD and hBD-2 mutant were calculated using the VMD program with the heavy atom distance cutoff of 3.0 Å and the angle cutoff of 20° deviation from H-bond linearity. The time a particular H-bond is formed over the course of the simulation is monitored and is expressed as % occupancy. In order to find out the residues on the binding interface, the closest distance between every residue atom (including hydrogen) between the RBD and hBD-2m was calculated and averaged over the trajectory run. The average distances between each residue on RBD and on hBD-2m are shaded by proximity on a red to white color-scale and were used to build the distance maps.

The MM-GBSA method (Genheden and Ryde, 2015) was applied to calculate the binding interaction energy for hBD-2 mutant with RBD. Due to limitations in estimating entropic contributions, MM-GBSA predictions were used to assess relative binding interaction trends among the studied peptide–protein complexes, rather than absolute binding affinities. The total pairwise interaction energy (*E*_*binding*_) was calculated over the entire trajectory by NAMD energy plugin of the VMD using Equation 3:

$$E_{binding}=<E_{complex}>-<E_{RBD}>-<E_{hBD-2m}>$$ (3)

*E*_*complex*_ is the potential energy of hBD-2m-RBD complex, *E*_*hBD-2m*_ is the potential energy of hBD-2m, and *E*_*RBD*_ is the potential energy of RBD. < > is the ensemble average over simulation time. In the MM-GBSA method, the solvent effect was counted using the generalized Born implicit solvent model (GBIS) (Tanner et al., 2011), (He et al., 2020). The NAMD trajectories were output with a frequency of 5 ps with the length of 500 ns each. So each trajectory has 100,000 frames, and 100,000 datapoints were sampled in the MM-GBSA calculation.

## 3 Results

### 3.1 Results of hBD-2 block-mutant binding with RBD

Building on the systematic point-mutation results obtained in our previous work (Jahan and Zhang, 2025), we explored multiple-site mutations (hereafter referred to as block-mutations) within RES18-30 region of hBD-2 to identify sequences with enhanced binding affinity with the RBD. The sequences of the 32 block-mutants are listed in Supplementary Table S1. The objective was to identify mutants with interaction energies lower than that of the wildtype peptide. After predicting the structures of the hBD-2 block-mutants using AlphaFold2, molecular docking was performed using HADDOCK 2.4 for all 32 block-mutants as well as the wildtype peptide to predict initial binding structures with the RBD. Subsequently, molecular dynamics simulations of 500 ns were performed for each complex following the same protocol described in our previous work (Jahan and Zhang, 2025) with the details shown in the Materials and Methods section. Structural stability was assessed through RMSD analysis (Supplementary Table S2). Based on RMSD values calculated, the resulting files were processed with custom Python Script using Seaborn (kdeplot) and Matplotlib to generate the KDE probability RMSD density plots (Figures 3a-d). The binding interaction energies were calculated using GMX/MMPBSA based on the last 100 ns of each simulation (Figure 4). The corresponding results are summarized in Supplementary Table S2 in SI.

**FIGURE 3:**
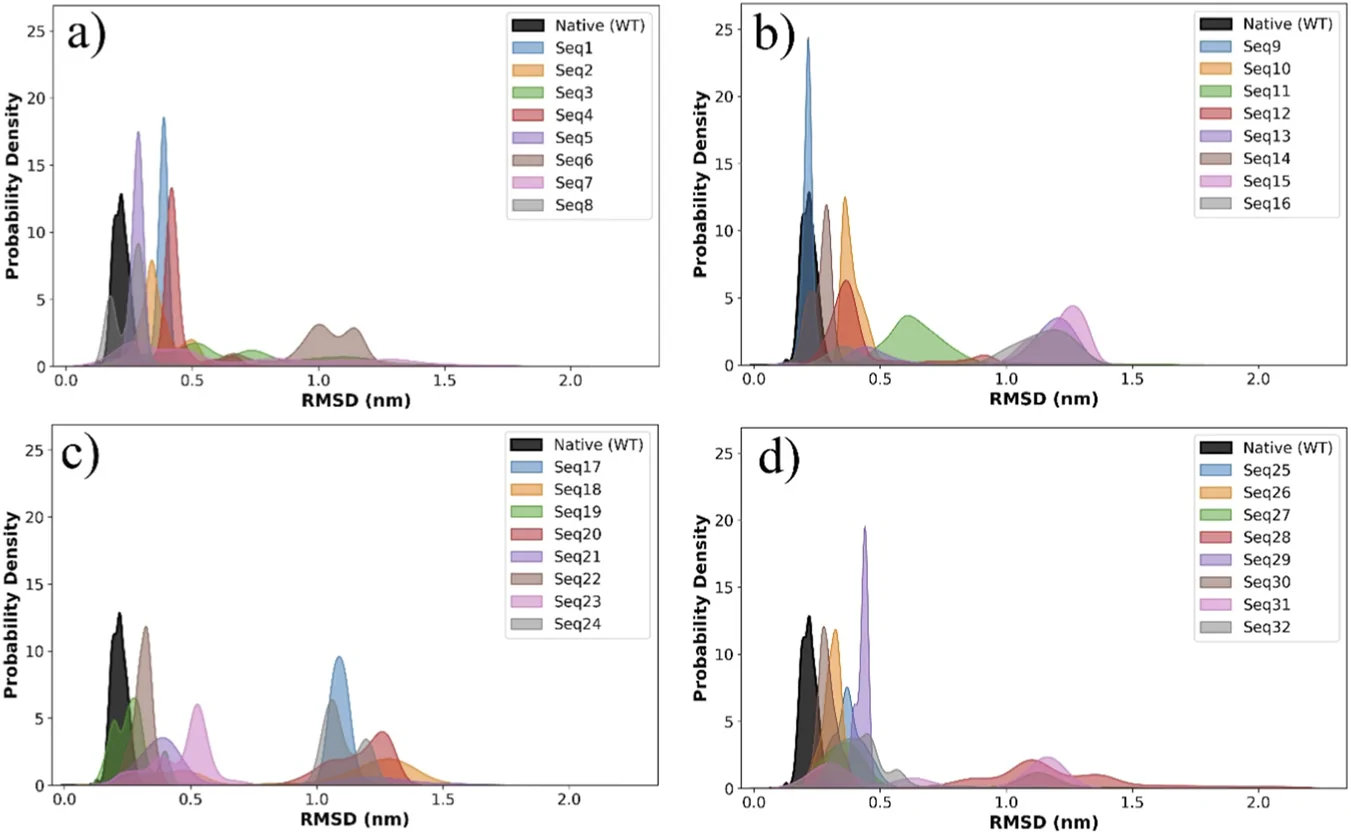
The RMSD probability density of different hBD-2 block-mutants bound with RBD simulations in comparison to the RBD&hBD-2 wildtype based on Gromacs simulation trajectories.

**FIGURE 4:**
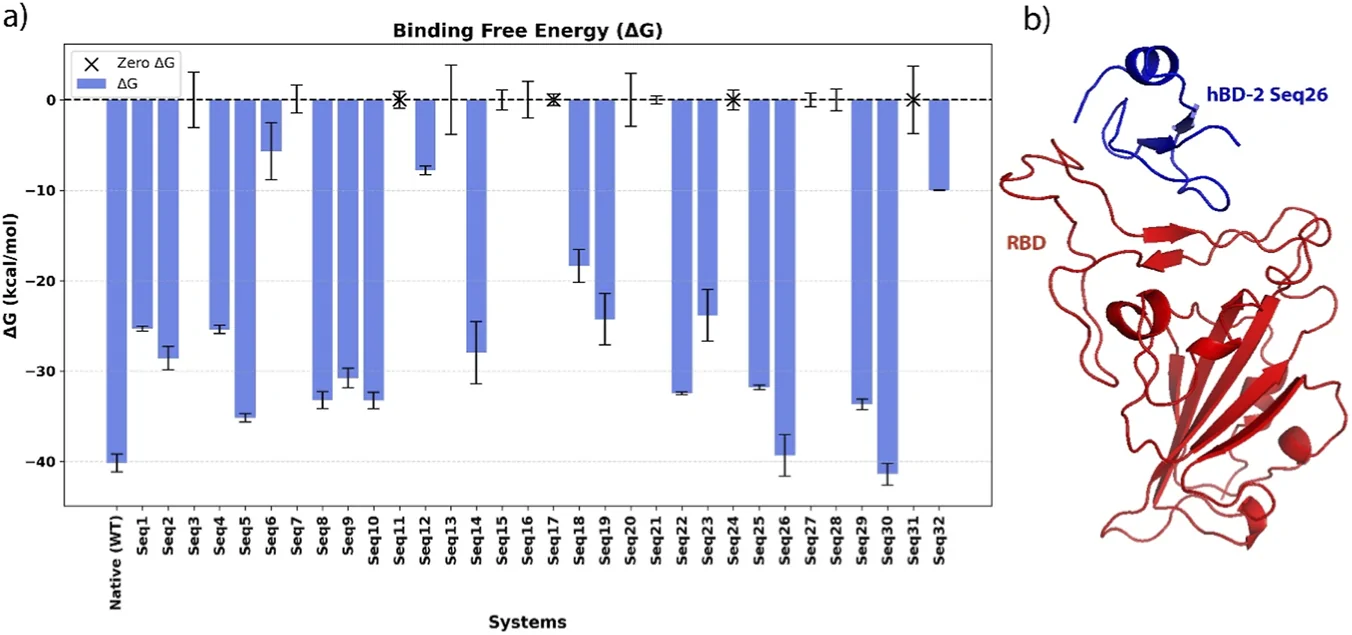
The free energy comparison of 32 hBD-2 multiple-site mutants binding with RBD in comparison with hBD-2 wildtype bound with RBD **(a)** based on Gromacs simulations, and the stable binding structure of RBD with block-mutant Seq26 after 500 ns NAMD simulations **(b)** which has RBD shown in red while hBD-2 block-mutant in blue cartoon.

As shown in Supplementary Table S2, the average RMSD values of several complexes were significantly higher than that of the wildtype complex, reaching up to 11.8 Å compared with 2.2 Å for the wildtype. The RMSD distribution profile shown in Figure 3 suggests that hBD-2 dissociated from the RBD in these cases. As a result, the calculated binding free energy (ΔG) values during the last 100 ns of the simulations were close to zero as shown in Figure 4. Among the various sequence combinations examined, Seq30 exhibited a lower interaction energy than the wildtype, whereas Seq26 showed an interaction energy comparable to that of the wildtype. However, the average number of hydrogen bonds observed for Seq30 and Seq26 were lower than that of the wildtype, suggesting that hydrogen bond interactions are not necessarily the primary driving force governing the binding between hBD-2 mutants and RBD.

Considering the binding free energy, RMSD, and hydrogen bonding results collectively, Seq5, Seq8, Seq10, Seq22, Seq26, Seq29 and Seq30 emerged as promising candidates. In order to further evaluate the stability of these seven hBD-2 mutants in complex with RBD, independent replicate simulations were performed using NAMD, starting from the same initial structures used in the original simulations. The RMSD of the protein complex, the number of hydrogen bonds, the binding interaction energy, and the buried surface area (BSA) between hBD-2 and RBD during the 500 ns simulations are summarized in Supplementary Table S2. Among the seven candidates, only Seq26 exhibited binding interaction energies, complex RMSD values, number of hydrogen bonds, and BSA results from the replicate simulation that were consistent with those obtained in the original simulation. Comparisons of the initial complex structure, the final complex structures from the original run and the rerun, the result is shown in Figure 5a. Except the flexible loop region and the head of hBD-2, other regions are very consistent in the complex structures between the original and rerun simulations. The RMSD of the RBD&hBD-2 complex, the hBD-2 mutant, and the RBD during in the rerun are shown in Figure 5b. Throughout the 500 ns rerun, both the complex and the individual protein in the complex are stable structurally. The RMSF of both hBD-2 and RBD are shown in Figure 5c, which are similar to those reported previously for the wildtype hBD-2 and RBD in our previous work (Zhang et al., 2022). Analyzing the number of hydrogen bonds formed between RBD and hBD-2 mutant of Seq26, the original simulation has an average number of hydrogen bonds of 1.54, and a similar number in rerun (1.53). The change of hydrogen bond number over simulation time for the rerun is shown in Figure 5d. Examination of the residue pairs involved in hydrogen-bond formation indicated that Lys36 on hBD-2 mutant (or abbreviated as hBD-2m) and Glu484 on RBD, Pro21 on hBD-2m and Ser494 on RBD, Arg29 on hBD-2m and Val483 and Glu484 on RBD, Gln26 on hBD-2m and Gln493 on RBD, Tyr24 on hBD-2m and Gln493 on RBD form stable hydrogen bonds in both the original run and the rerun.

**FIGURE 5:**
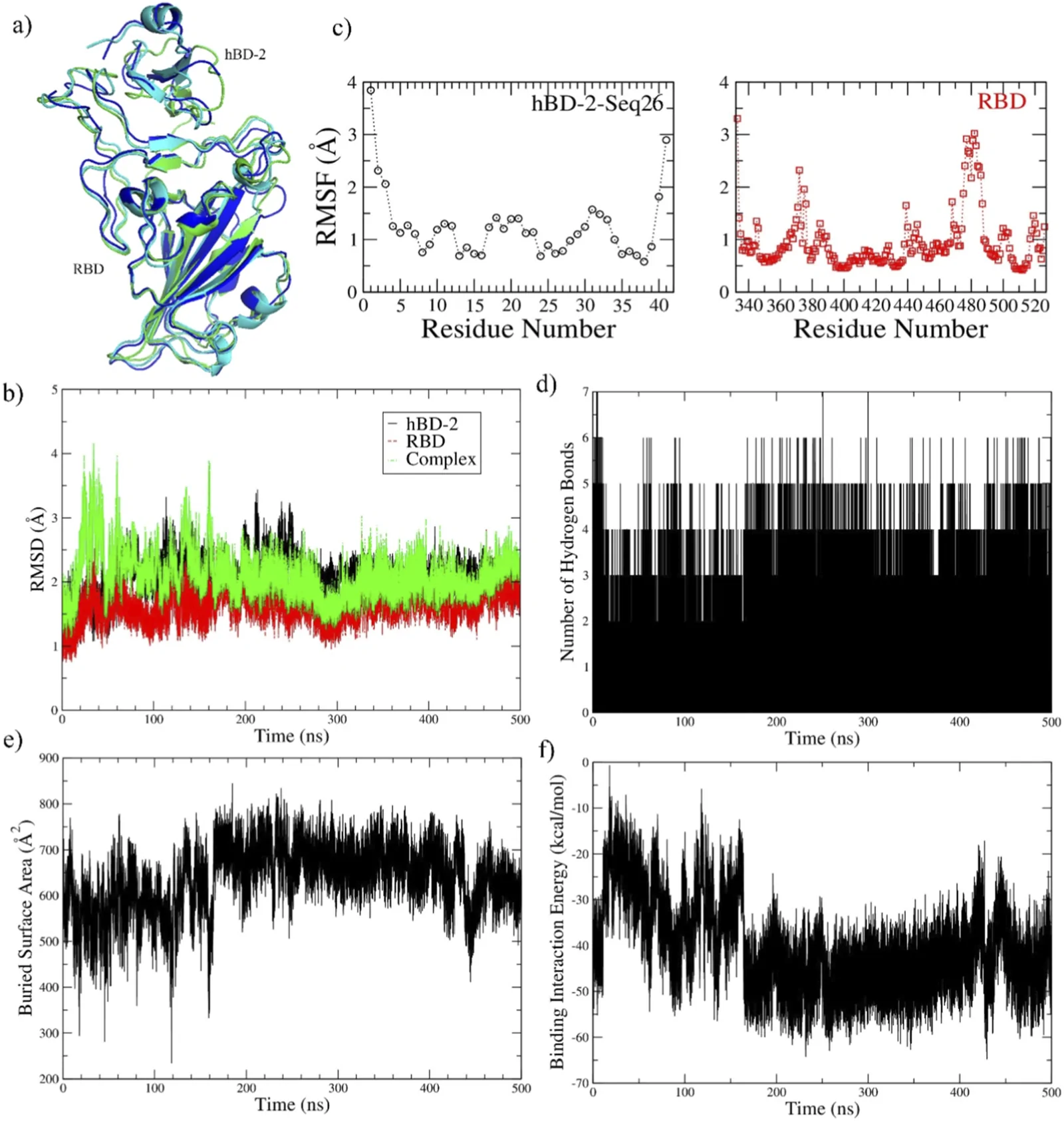
The comparison of the initial (green) and last structures of RBD&hBD2-Seq26 after 500 ns Gromacs simulations in the original (cyan) and 500 ns NAMD rerun (blue) simulations **(a)**, the RMSD **(b)**, RMSF **(c)**, the number of hydrogen bonds formed **(d)**, buried surface area **(e)** and binding interaction energy **(f)** between RBD and hBD-2 block-mutant Seq26 during 500 ns NAMD rerun simulation for block-mutant Seq26 bound with RBD.

Calculating BSA and binding interaction energy between the RBD and hBD-2m, the results of Seq26 during 500 ns NAMD simulations are shown in Figure 5e,f. Besides that, the block average of binding interaction energy is shown in Supplementary Table S1a. Those prove that during the last 200 ns of the 500 ns NAMD simulation, the hBD-2 mutant remained stably bound to RBD, indicating a robust protein-protein interaction.

The distance map calculated for Seq26-RBD complex is shown in Figure 6a. The binding pattern is highly similar to that observed for the wildtype hBD-2-RBD complex reported in our previous work (Zhang et al., 2022). These results indicate that the hBD-2-Seq26 mutant retains binding with RBD at the RES18-RES30 region of hBD-2. Overall, the simulation results suggest that Seq26 is a promising hBD-2 mutant capable of forming a stable complex with RBD.

**FIGURE 6:**
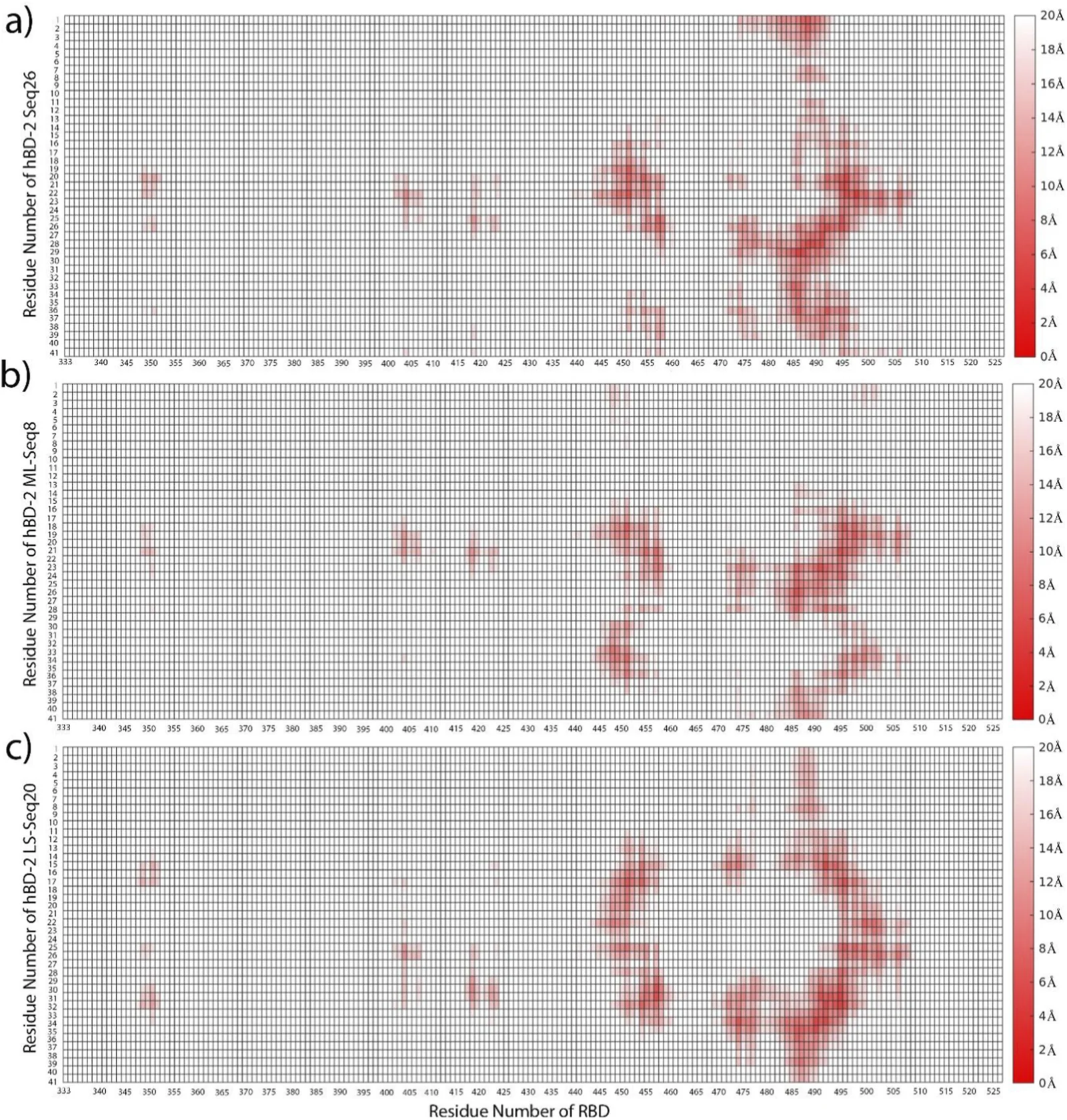
Comparison of distance maps of hBD-2 of block-mutant Seq26 **(a)**, ML-Seq8 **(b)**, and LS-Seq20 **(c)** bound with RBD based on NAMD simulation results.

### 3.2 CNN model performance analysis

Based on the simulation results obtained for the 247 hBD-2 spot-mutants in complex with RBD from our previous work (Jahan and Zhang, 2025), together with the results for the hBD-2 block-mutants summarized in Supplementary Table S2, the first AI model, CNN model was trained.

The CNN model was trained for 100 epochs, during which a consistent improvement in predictive performance was observed. Supplementary Figure S2 presents the evolution of Root Mean Square Error (RMSE) and Mean Absolute Error (MAE) for both the training and validation datasets. As shown in the figure, both RMSE and MAE gradually decreased throughout the training process. Notably, the training and validation curves exhibited similar patterns, suggesting that the model generalized well to unseen data and showed minimal evidence of overfitting. Table 1 summarizes the final performance metrics obtained for the training and test datasets.

**TABLE 1:** CNN model performance metrics.

Metric	Training set	Test set
MAE	5.4041	5.8275
RMSE	7.1813	7.6073
R^2^ score	0.7296	0.6872

The model achieved comparable MAE, values for both datasets, with values of 5.4041 and 5.8275 for the training and test sets, respectively. Similarly, the RMSE, values of 7.1813 and 7.6073 for the training and test sets, respectively, further demonstrate the model’s consistent predictive performance. The R^2^ values of 0.7296 for the training set and 0.6872 for the test set indicate that our model accounts for a substantial proportion of the variance in the predicted ΔG, values.

These results demonstrate the potential of the CNN-based approach for predicting ΔG changes associated with mutations at the 13 target residues of hBD-2. However, the R^2^ values also indicate that a portion of the variance remain unexplained, suggesting that further model refinement may be required to capture additional factors influencing ΔG prediction. Such improvements may be achieved through the incorporation of larger datasets and additional experimental data.

### 3.3 LSTM model performance analysis

Based on the simulation results from the 247 spot-mutants of hBD-2 [20], and 32 block-mutants of hBD-2 binding with RBD as results shown in Supplementary Table S2, the LSTM model was trained as well.

Similar to CNN model, the LSTM model’s training process, conducted over 100 epochs, also revealed a consistent improvement in predictive accuracy. Supplementary Table S3 presents the evolution of Root Mean Square Error (RMSE) and Mean Absolute Error (MAE) for both training and validation datasets.

The graphical representation illustrates a gradual reduction in both RMSE and MAE as the training progressed. Notably, the trajectories of the training and validation curves exhibit similar patterns, suggesting that the model maintains its predictive power when faced with unseen data, thus indicating minimal overfitting. Table 2 shows the final performance metrics for the training and test sets.

**TABLE 2:** LSTM model performance metrics.

Metric	Training set	Test set
MAE	5.3064	4.4320
RMSE	7.0040	5.7474
R^2^ score	0.7411	0.8276

The model achieved comparable MAE, values across both datasets: 5.3064 for training and 4.4320 for testing. Similarly, the RMSE, values of 7.0040 and 5.7474 for training and test sets respectively further corroborate the model’s consistent performance. The R^2^ scores, 0.7411 (training) and 0.8276 (test), indicate that the model accounts for a significant portion of the variance in ΔG, values.

These results demonstrate the potential of our LSTM-based approach in predicting ΔG values for protein sequences modified with 13 residues of hBD-2. While the model shows promise, the R^2^ scores suggest there is still unexplained variance, pointing to opportunities for further model refinement to capture additional complexities in prediction of ΔG value with more experimental dataset.

### 3.4 Assessing the structural stability of RBD&hBD-2-mutant complexes with hBD-2-mutant sequences predicted from ML

CNN Prediction Results. Based on the trained AI model, 30 hBD-2-mutant sequences were predicted. For each mutant, the three-dimensional structure was generated using AlphaFold2 program, followed by HADDOCK docking to predict the most favourable binding conformation. The AlphaFold2 prediction scores for these hBD-2 mutants are summarized in Supplementary Table S5 in SI. For each mutant, the docked complex with the lowest HADDOCK score was selected for subsequent GROMACS molecular dynamics (MD) simulations for 500 ns

The gmx-MM/PBSA package was applied to calculate the binding free energy of each hBD-2 mutant in complex with RBD. The average binding free energy (ΔG) and corresponding standard deviations are shown in Figure 7a, with the numerical values summarized in Supplementary Table S6. Except for ML6 and ML29, most hBD-2 mutants can bind with RBD stably during 500 ns GROMACS simulations. Notably, the ML3-RBD, ML13-RBD, and ML24-RBD complexes exhibited ΔG values comparable to that of the wildtype hBD2-RBD complex, and ML8-RBD displayed slightly higher ΔG values than the wildtype; whereas ML7 and ML20 displayed lower ΔG values than the wildtype, indicating stronger binding interactions and greater complex stability.

**FIGURE 7:**
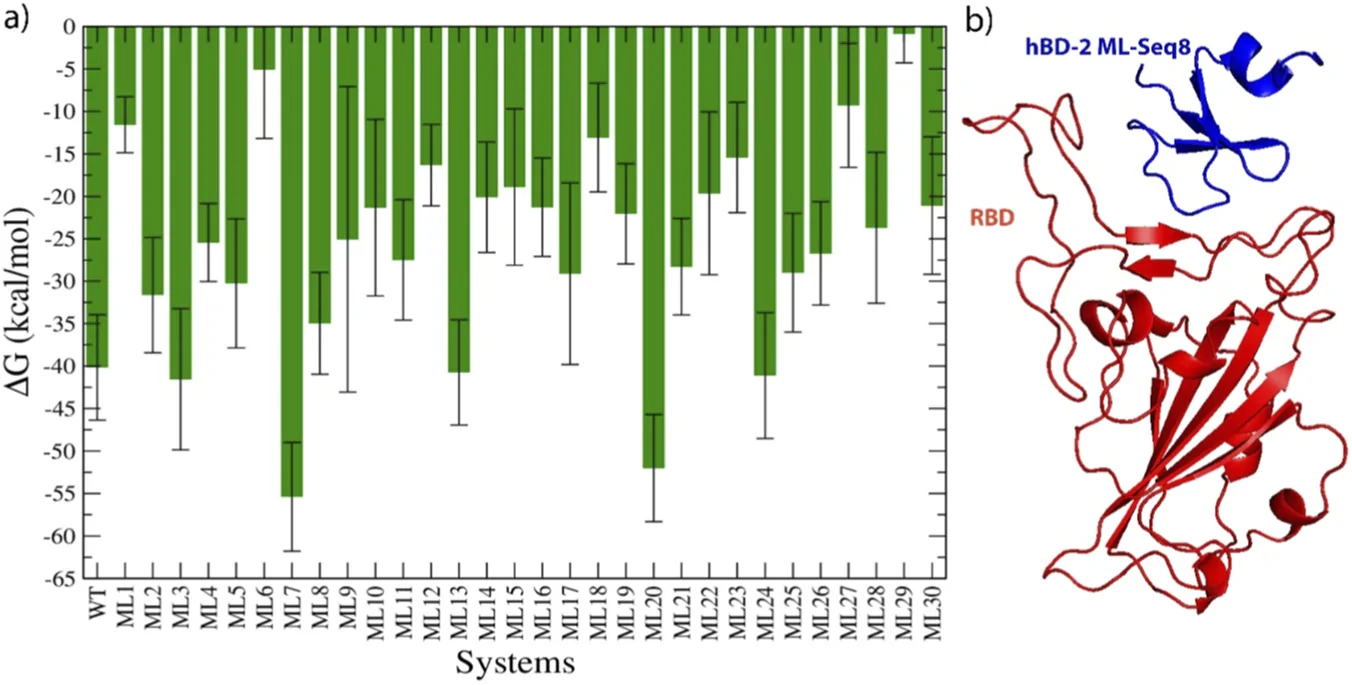
Binding interaction energy and standard deviation comparison on hBD-2 mutants with sequences predicted from ML binding with RBD **(a)**, and the stable binding structure of hBD-2-ML-Seq8 with RBD predicted from rerun simulation **(b)**.

Besides the binding interaction energy, the average backbone RMSD of each complex structure was analyzed and is reported in Supplementary Table S6. Among the 30 complexes examined, 17 exhibited average RMSD values below 5.0 Å, indicating stable binding between the hBD-2 mutants and RBD. Another 9 complexes showed RMSD values between 5.0 to 10.0 Å, whereas only 4 complexes exhibited RMSD values of approximately 10 Å or higher, indicating substantial structural deviations from the initial conformations. These results suggest that most mutations contribute to maintaining or enhancing complex stability, whereas only a small subset of mutants destabilize the complex. Based on the RMSD analysis, ML2, ML3, ML5, ML7, ML8, ML13, ML20, and ML24 exhibited structural stability comparable to that of the wildtype hBD-2-RBD complex.

To further characterize the binding interactions, the average number of hydrogen bonds formed during the final 100 ns of each simulation was calculated (Supplementary Table S6). The results indicated that ML3 and ML24 formed more hydrogen bonds with the RBD than the wildtype complex. This observation is consistent with the binding free energy analysis and further supports the presence of stronger molecular interactions. In contrast, although ML7, ML13, and ML20 exhibited significantly lower binding free energies than the wildtype, they formed fewer hydrogen bonds. These findings suggest that hydrogen bonding is not the sole determinant of binding strength and that other intermolecular interactions contribute substantially to complex formation and stability.

To validate the simulation results, rerun simulations were performed for eight selected mutants, namely ML2, ML3, ML5, ML7, ML8, ML13, ML20, and ML24, based on their favourable RMSD and binding free energy values (highlighted in Supplementary Table S6). Among these eight candidates, only ML3, ML5, and ML8 still have favourable binding interactions with RBD in the rerun. Comparisons of the binding structures of ML3, ML5, and ML8 with the wildtype hBD-2-RBD complex are presented in Supplementary Figure S4, while the corresponding distance map comparisons are shown in Supplementary Figure S5. Overall, all three mutants bound with RBD at a binding interface similar to that observed for the wildtype complex. However, a slight rotation of ML8 toward RBD relative to the initial structure was observed (Supplementary Figure S4d). All three complexes remained stable during both the original and rerun simulations, as demonstrated by the RMSD profiles shown in Supplementary Figure S6(Left), and exhibited similar BSA at the binding interface (Supplementary Figure S6(Right)). Interestingly, the number of hydrogen bonds formed at the ML8-RBD (or ML-Seq8-RBD) interface exhibited an increasing trend over time (Supplementary Figure S7). Furthermore, ML8 displayed favourable binding free energies in both the original and rerun simulations. When considered together with the structural stability analyses, these results identify ML8 as the most promising CNN-predicted hBD-2 mutant. The representative stable binding structure of ML8-RBD complex is shown in Figure 7b.

The comparisons between the initial and final structures, RMSF, RMSD, number of hydrogen bonds formed, BSA, and binding interaction energy results for ML8-RBD are shown in Figures 8a–f, respectively. The block average of binding interaction energy in rerun simulation is shown in Supplementary Figure S1b. The corresponding distance map is presented in Supplementary Figure S6b. Interestingly, the hBD-2 mutant ML8 and RBD became nearly dissociated at around 50–60 ns during the simulation. Then the two proteins reoriented toward the correct binding interface, resulting in a progressively more stable complex over the remainder of the simulation. Consequently, the final structure obtained from the rerun differs from the initial structure and exhibits a moderate deviation from the final structure obtained in the original simulation. Nevertheless, the RMSD analysis indicates that both the complex and the individual protein components remained stable from 100 ns to 500 ns? These results suggest that ML8 forms a stable complex with RBD and is capable of achieving a lower binding interaction energy than the wildtype hBD-2 complex.

**FIGURE 8:**
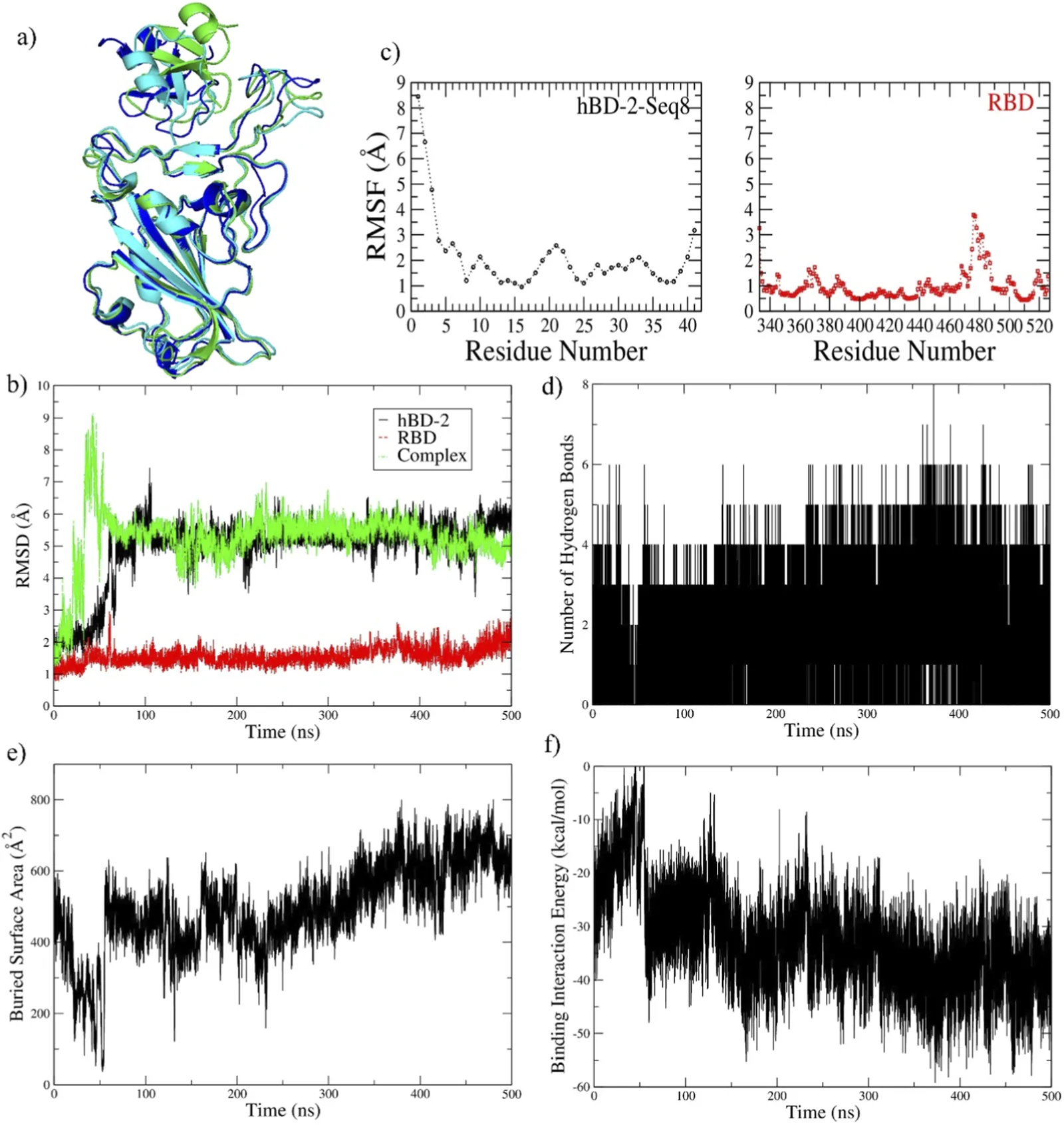
The comparison of the initial (green) and last structures of RBD&hBD2-ML8 predicted from the original (cyan) and rerun (blue) simulations **(a)**, the RMSD **(b)**, RMSF **(c)**, the number of hydrogen bonds formed **(d)**, buried surface area **(e)** and binding interaction energy **(f)** between RBD and hBD-2-ML8 during 500 ns NAMD rerun simulation.

LSTM Prediction Results. Using the complete dataset, including the MD simulation results summarized in Supplementary Table S6, another 20 hBD-2 mutant sequences were predicted from LSTM model. The mutated sequences within RES18-RES30 regions are listed in Supplementary Table S7. Following the same steps conducted on the CNN model predicted mutants, AlphaFold2 program was applied to predict the structures of hBD-2 mutants, and HADDOCK docking was subsequently performed to predict the binding interfaces between the hBD-2 mutants and RBD. All-atom MD simulations were then conducted for the resulting complexes. The binding interaction energies, RMSD and BSA of complexes, and number of hydrogen bonds formed between the hBD-2 mutants and RBD were calculated and summarized (Supplementary Table S7). A comparison of the binding interaction energies is presented in Figure 9a, which identified three mutants, namely LS-Seq11, LS-Seq14, and LS-Seq20 as particularly promising because their predicted binding energies were lower than those of the stable mutants reported in Supplementary Table S6 (analyzed above). To validate these results, repeat runs were performed on the three selected mutants starting from the same initial configurations using NAMD simulations. The rerun simulations for all three mutants remained stable, as demonstrated by the RMSD vs. simulation time results for LS-Seq11, LS-Seq14 and LS-Seq20 shown in Supplementary Figure S8. The complex structures remained stable in both the original and rerun simulations, as the comparison of the complex structures in LS-Seq11, Seq14 and Seq20 jobs presented in Supplementary Figure S9. These results suggest that all three hBD-2 mutants are capable of forming stable complexes with RBD. All three hBD-2 mutants bind on RBD within the correct region; however, the exact binding interfaces differ among the mutants. The representative stable structure of the LS-Seq20-RBD complex is shown in Figure 9b. While LS-Seq20 still formed a strong binding with RBD in the rerun by displaying a similarly low binding interaction energy to the original run, LS-Seq11 and LS-Seq14 had weaker binding with RBD than the original runs, as the binding interaction energies obtained from the repeat simulations reported in Supplementary Table S7.

**FIGURE 9:**
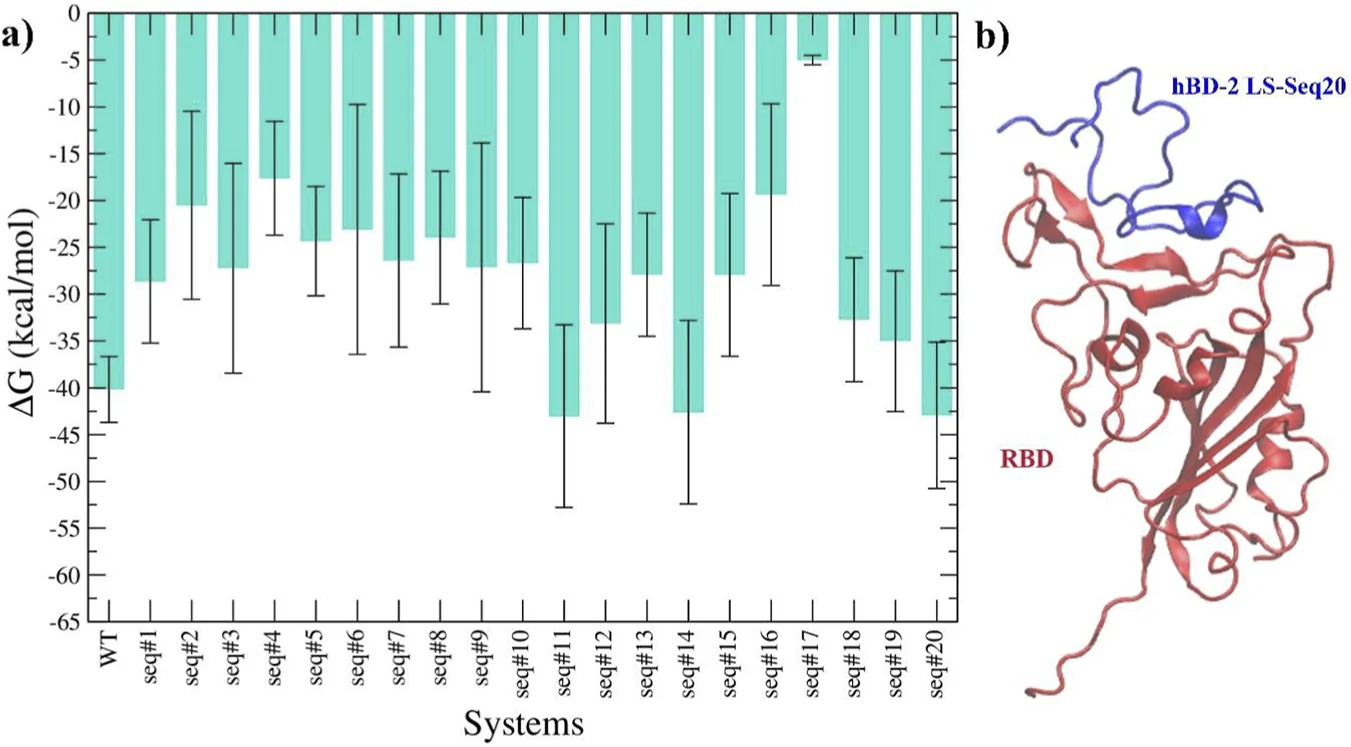
Binding interaction energy and standard deviation comparison on hBD-2 mutants with sequences predicted from 20 LSTM model binding with RBD **(a)**, and the stable binding structure of hBD-2-LS-Seq20 with RBD predicted from NAMD simulation **(b)**.

Among the three candidates, LS-Seq20 emerged as the most promising mutant, exhibiting a gradual decreases in RMSD over time and only minor structural deviations between the initial and final conformations. Consequently, the rerun simulation of the LS-Seq20-RBD complex was extended to 600 ns The comparisons between the initial and final structures, the RMSF profiles of hBD-2 mutant and RBD, the RMSD values of the complex and its individual components, the number of the hydrogen bonds formed, BSA, and the binding interaction energy during the 600 ns NAMD simulation are shown in Figures 10a–f, respectively. They all prove the complex is stable over time. A small rotation of the hBD-2 mutant at the binding interface was observed during the simulation (Figure 10a). This conformational adjustment appears to stabilize the interaction between hBD-2 mutant and RBD, as reflected by the favourable binding interaction profile shown in Figure 10f. The block average of binding interaction energy in rerun simulation is shown in Supplementary Figure S1c, which also indicates the stable binding of LS-Seq20 and RBD over time. The corresponding distance map is presented in Figure 6c, which overall agrees with the wildtype result with slight shifting.

**FIGURE 10:**
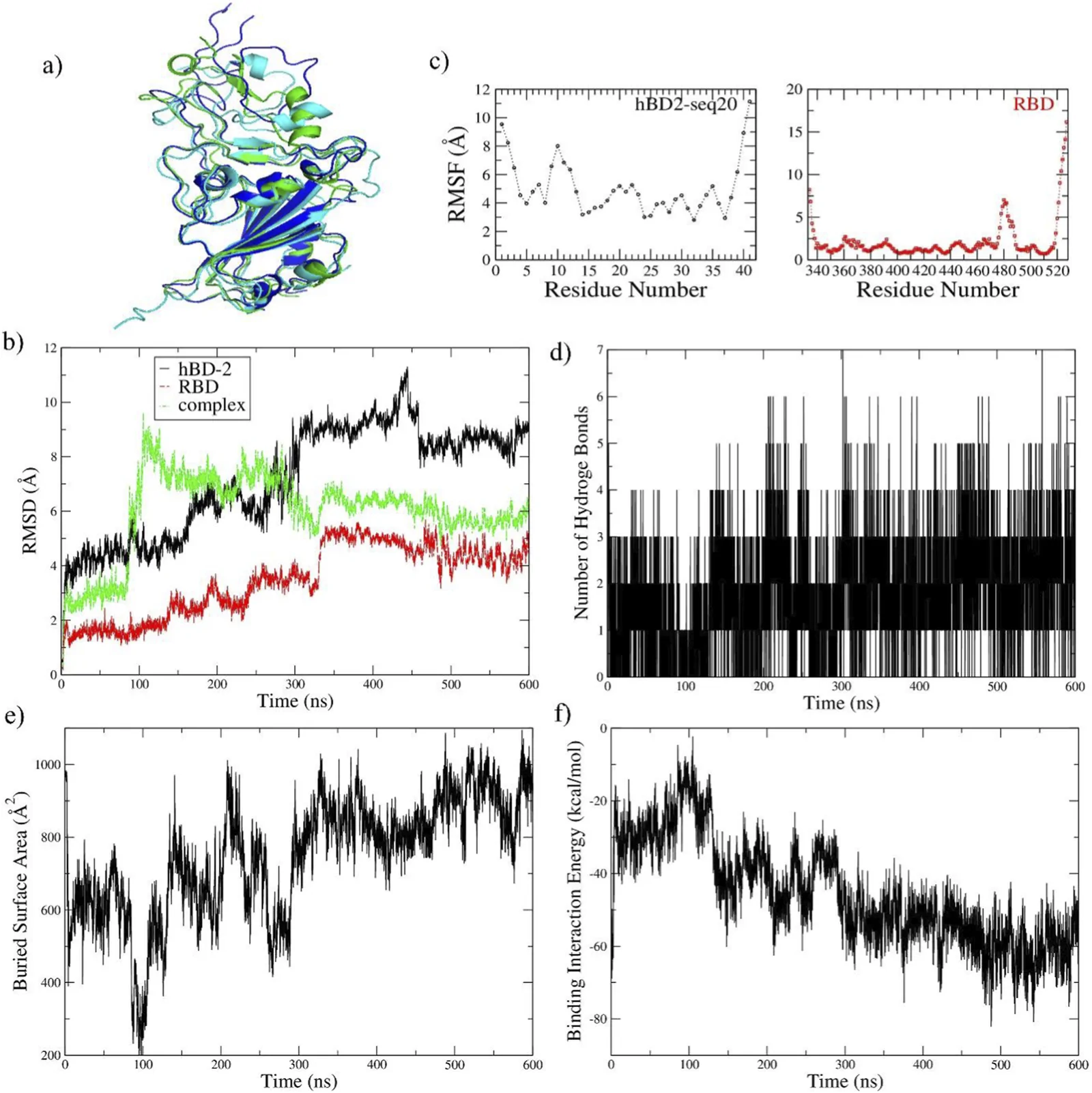
The comparison of the initial (green) and last structures of hBD-2 LS-Seq20 bound with RBD based on the original (cyan) and rerun (blue) simulations **(a)**, the RMSD **(b)**, RMSF **(c)**, the number of hydrogen bonds formed **(d)**, buried surface area **(e)** and binding interaction energy **(f)** between RBD and hBD-2 LS-Seq20 during 600 ns NAMD simulation.

ADMET Prediction Result. Based on the sequences of hBD-2 wildtype and three novel candidates, two online programs were applied to predict the ADMET properties as result shown in Table 3. The net charge, hydropathicity, PI, and instability index were predicted from ProtParam online program (Gasteiger et al., 2005), while hydrophobicity, hydrophilicity and toxin were predicted by ToxinPred3.0 program (Rathore et al., 2024).

**TABLE 3:** Physicochemical properties of hBD-2 wildtype and novel candidates predicted.

Peptide	Charge	Hydropathicity	PI	Instability index	Hydrophobicity	Hydrophilicity	Toxin
Seq26	+5	−0.063	9.23	19.24 (stable)	−0.09	−0.25	Toxin
ML-Seq8	+6	−0.115	9.50	36.29 (stable)	−0.12	−0.01	Toxin
LS-Seq20	+6	−0.351	9.62	33.52 (stable)	−0.15	0.10	Toxin
Wildtype	+6	−0.102	9.30	42.30 (unstable)	−0.14	−0.01	Toxin

Both the hBD-2 wildtype and the novel candidates have the same sequence length, with each peptide consisting of 41 residues. However, they differ slightly in amino acid composition because the novel candidates contain mutations within the RES18-RES30 region. According to predictions from two online programs, the novel candidates have a net positive charge (+5 to +6) that is equal to or very similar to that of the wildtype (+6), and they exhibit a toxicity profile comparable to that of the wildtype. Consequently, both the wildtype and the novel candidates are predicted to be toxic.

According to ToxinPred3.0 prediction, a more negative hydrophobicity value indicates that a peptide is less hydrophobic (i.e., more hydrophilic). As shown in Table 3, LS-Seq20 has the lowest hydrophobicity and the highest hydrophilicity among the four peptides, and therefore exhibits the lowest overall hydropathicity. The hBD-2 wildtype has a higher instability index value than all of the novel candidates, and is predicted to be unstable under *in vitro* conditions. In contrast, all the novel candidates have lower instability index values than the wildtype, suggesting greater predicted *in vitro* stability. These results indicate that the designed peptide candidates possess improved sequence-based stability compared with the hBD-2 wildtype.

## 4 Discussion

### 4.1 Method discussion

Comparison of CNN, LSTM, and literature-based design. In this project, both CNN and LSTM models were applied to predict the hBD-2 mutant sequences with improved binding stability relative to the wildtype. Based on the 30 CNN-predicted mutants (Supplementary Table S6), the average binding interaction energy is −25.53 kcal/mol, whereas for the LSTM-predicted mutants (Supplementary Table S7), the average is −27.75 kcal/mol. Overall, the LSTM model outperforms the CNN model. For the 32 block-mutants (Supplementary Table S2), the average binding interaction energy is −15.88 kcal/mol. Thus, the LSTM predictions are the most accurate, while the block-mutant designed–based on spot-mutation data–performs the worst in terms of binding energy prediction, likely due to increases stochasticity in the design process. In terms of structural stability, the average RMSD values of hBD-2m-RBD complexes are 6.12 Å for block-mutants, 5.53 Å for CNN-generated mutants, and 5.23 Å for LSTM-generated mutants. Overall, the LSTM predicted mutants form slightly more stable complexes with RBD than the CNN-predicted mutants and significantly outperform the block-mutant designs.

Beyond the MD simulation results, the superiority of the LSTM model is also evident at the level of predictive performance. As shown in Tables 1, 2, on the held-out test set the LSTM achieves an RMSE of 5.7474 kcal/mol for ΔG prediction, compared with 7.6073 kcal/mol for the CNN, corresponding to an approximately 24% reduction. Similarly, the LSTM test MAE of 4.4320 is about 24% lower than the CNN MAE of 5.8275, while the LSTM test R^2^ of 0.8276 is substantially higher than the CNN value of 0.6872, indicating that the LSTM explains approximately 83% of the variance in MD-derived ΔG values compared with 69% for the CNN. The lower RMSE indicates that LSTM predictions are closer to MD-derived reference values, which is consistent with its improved performance in identifying sequences with favorable binding energies.

The performance gap can be attributed to differences in model architecture. The CNN, using Conv1D layers with a kernel size of 3, captures primarily local sequence motifs within short sliding windows and is therefore limited in modelling long-range residue-residue interactions across the 13-residue mutation region (RES18-RES30). In contrast, the LSTM processes sequences through gated memory cells (input, forget, and output gates), enabling it to retain long-range dependencies along the full peptide sequence and capture how mutations at distant positions influence binding. Furthermore, the LSTM employs a learned 10-dimensional embedding layer instead of raw integer encoding, allowing it to learn chemically meaningful representations of amino acids, including properties such as hydrophobicity, polarity, and charge. The model also incorporates dropout regularization (p = 0.2) in the fully connected layers, reducing overfitting and improving generalization. Finally, the LSTM integrates sequence and simulation time information through a fully connected architecture (65 → 32 → 1), making it better suited for modeling time dependent ΔG variations in MD trajectories. Together, these features explain the superior predictive accuracy and improved downstream sequence design performance of the LSTM model.

In summary, AI-based prediction of mutant peptide sequences provides a promising strategy for computational drug design, and in this study, the LSTM model demonstrates superior performance compared with the CNN model.

Limitations of training data size. We acknowledge that training deep learning models on 279 total sequences (247 spot-mutants and 32 block-mutants) carries an inherent risk of overfitting for architectures such as CNN and LSTM. To mitigate this, we applied dropout regularization (p = 0.2) in the LSTM’s fully connected layers, and we observed close agreement between training and test performance metrics for both the CNN (Table 1) and LSTM (Table 2) models, suggesting the models generalize reasonably well rather than memorizing the training set. Nonetheless, small training sets remain a limitation for deep learning models applied to biomedical sequence data, and recent work on data augmentation, cross-validation, and transfer learning strategies for small biomedical datasets (Rathore et al., 2024) offers concrete directions for improving model robustness as more molecular dynamics simulation data becomes available.

AlphaFold2 prediction. We predicted the secondary structure using Alphafold2 for block-mutants, CNN model predicted mutants and LSTM model predicted mutants and reported the plDDT scores and pTM scores in Supplementary Table S1, S5 and S7, respectively. Based on the data, the average plDDT score is 78.42 and the average pTM score is 0.504 for block-mutants, while 67.64 and 0.391 for CNN model predicted mutants, and 77.985 and 0.493 for LSTM model prediction. The higher the plDDT score, the better the local accuracy of each residue; and the higher the pTM score, the better the overall accuracy of the global fold. Since these plDDT scores are either close to 80 or at least close to 70, the secondary structures of hBD-2 mutants predicted from Alphafold2 should be confident (ranking is high). Also, although the average pTM score for CNN model is lower than 0.5, the average pTM scores for block-mutants and LSTM are very close to 0.5. Thus the overall fold should be correct. Thus, it is OK to trust the secondary structure predicted from Alphafold2 and use them as initial input for following HADDOCK docking and MD predictions.

### 4.2 AMP prediction discussion

Sequence comparison between novel candidates and wildtype. In this project, in total 32 block-mutants, 30 CNN-predicted mutants, and 20 LSTM-predicted mutants were designed. The block-mutants were systematically generated by introducing multiple substitutions within RES18-RES30 of hBD-2 simultaneously, guided by combinations of 11 previously identified beneficial point-mutations that enhance hBD-2-RBD binding (Jahan and Zhang, 2025). Among the block mutants, Seq26 exhibited binding interaction energies comparable to that of the wildtype hBD-2-RBD complex in both the original and rerun simulations and emerged as the promising candidate.

Using the native hBD-2 sequence within RES18-RES30 as a reference, additional AI-based modelling was performed to predict the optimized sequences with improved binding affinity with RBD. Two promising mutants generated by AI-based modelling, ML-Seq8 and LS-Seq20, were identified. The amino acid sequences of RES18-RES30 for Seq26, ML-Seq8, LS-Seq20, and hBD-2 wildtype are compared as shown below.

hBD-2 wildtype: {*VFCPRRYKQIGTC*}

Seq26:     {*VFIPWRYHQIYRC*}

ML-Seq8:       {*MIMGKWCPGIRRQ*}

LS-Seq20:      {*APTNHAKFMKRTG*}

The sequences of the three mutants differ substantially, and the ML-predicted sequences are also highly divergent from the hBD-2 wildtype. The average number of hydrogen bonds formed between Seq26 and RBD is 1.54 (1.53 in rerun), compared with 3.46 for ML-Seq8 and 2.17 (1.78 in rerun) for LS-Seq20. The wildtype hBD-2 forms an average of approximately 3.1 hydrogen bonds with RBD. These results suggest that, for Seq26 and LS-Seq20, interactions other than hydrogen bonding likely play a dominant role in binding to the RBD.

Binding driving force comparison. We have incorporated the MM-PBSA energy decomposition analysis to investigate the driving force for the hBD-2 mutants from block-mutant and from AI-based model prediction binding with RBD. The results of some example mutants are shown in Supplementary Tables S9, S10, and in Supplementary Figure S10. For Seq26, the binding is primarily driven by favorable van der Waals interactions (ΔEvdw = −75.85 kcal/mol) and supported by non-polar solvation (ΔEnon-polar = −9.98 kcal/mol), while the electrostatic contribution is minimal (ΔEele = −1.61 kcal/mol) and polar solvation opposes binding (ΔEpolar = +48.12 kcal/mol). These results confirm that hydrophobic/van der Waals interactions, rather than hydrogen bonding, are the major contributors to complex stability. For ML-Seq8, both electrostatic interaction and van der Waals interactions are the major driving force, while polar solvation opposes the binding. For LS-Seq20, it was found that the electrostatic interaction and van der Waals interactions are the major driving force for the binding as well.

RMSF result comparison. The RMSF of hBD-2 in wildtype and mutant forms was calculated as shown in Figures 5c, 8c, 10c for Seq26, ML-Seq8 and LS-Seq20 respectively. They were compared with RMSF of hBD-2 in wildtype bound with RBD as shown in Supplementary Figure S11. The hBD-2 wildtype has the lowest RMSF among the four peptides studied, thus its structure is the least flexible when bound with RBD. Except the head region, Seq26, ML-Seq8 have low RMSF very close to the hBD-2 wildtype, while LS-Seq20 has higher RMSF than the other three in different regions, especially the head, tail and first loop (RES8-RES14) region. That suggests that LS-Seq20 binds stably with RBD on regions other than the head, tail and first loop, although its binding interaction energy is the lowest.

Binding interface comparison. When comparing the stable complex structures of different mutants bound with RBD (Figures 4b, 7b, 9b), it is evident that although the overall binding region on the RBD is conserved, the specific binding interfaces on hBD-2 mutants vary. This is consistent with the distance map comparisons (Figures 6a–c), and hydrogen bond number comparison, driving force comparison, and RMSF comparison results above, which indicate that while the RBD binding region remains similar, the precise interaction patterns on hBD-2m can vary. Interface analysis shows that both hBD2-Seq26 and the hBD-2 wildtype share a highly similar binding mode with RBD (Figure 6a), whereas ML-Seq8 and LS-Seq20 exhibit a slight rotation in their binding orientation relative to the wildtype. Thus, mutations in the RES18-RES30 region can modulate the binding interface of the complex, although designed mutants are predicted to exhibit enhanced binding affinity with RBD based on MD simulations.

### 4.3 Comparison of hBD-2 with other AMPs

Anti-viral feature comparison between hBD-2 and other AMPs. Besides hBD-2, other AMPs such as HD5 and LL-37 have been found to be promising inhibitors blocking the SARS-CoV-2 entry. However, these three AMPs block SARS-CoV-2 entry with different mechanisms. HD5 has been found to bind with ACE2 with high affinity, masking key residues involved in spike recognition and significantly reducing SARS-CoV-2 pseudovirus entry into ACE2-expressing cells (Wang et al., 2020). Li et al. found that both HD5 and LL-37 can bind with ACE2 through molecular docking, MD simulations, and MM-PBSA free-energy calculations. However, LL-37 exhibited stronger intermolecular interactions and lower binding free energies than HD5, suggesting a greater ability to compete with the viral spike protein for ACE2 binding (Li et al., 2021). Roth et al. did surface plasmon resonance (SPR) experiments on LL-37 and found that LL-37 act through widespread binding across multiple regions of Spike. Their study confirmed the direct binding of LL-37 to both the spike protein and the isolated RBD and demonstrated inhibition of the spike–ACE2 interaction (Roth et al., 2025). Comparing to HD5 and LL37, hBD-2 directly binds with RBD of the SARS-CoV-2 virus thus blocking the entry of the virus through both experiments and simulation studies (Zhang et al., 2022). The current project predicted novel hBD-2-based mutants, which are expected to have enhanced binding affinity with RBD than the hBD-2 wildtype.

### 4.4 Future work

Future experimental work and AI model. Based on this work, three mutants (Seq26, ML-Seq8 and LS-Seq20) are predicted to exhibit binding affinities comparable to that of the hBD-2 wildtype, and LS-Seq20 appears to have a stronger binding affinity than the wildtype.

In our previous work (Jahan and Zhang, 2025), in total 247 hBD-2 spot-mutants were built by mutating each residue in RES18-RES30 region from the current residue to the other 19 residues. Based on binding free energy calculations, 11 spot-mutants exhibited binding affinities comparable to or stronger than that of the hBD-2 wildtype. Combining these findings with the present study, in total fourteen sequences are identified as promising candidates with potential inhibitory activity against SARS-CoV-2 comparable to that of the hBD-2 wildtype. Experimental validation using microscale thermophoresis (MST) will be performed to determine the equilibrium binding affinities of the hBD-2 mutant peptides for the RBD. We also plan to conduct advanced solution NMR studies to characterize the molecular basis of their interactions with the RBD. The top-performing mutants validated by MST and NMR will be further evaluated in viral neutralization assays to assess their ability to neutralize SARS-CoV-2. These experimental results will also be applied to train AI models. Since RMSF can help predict the stable binding between protein and ligand (Famuyiwa et al., 2025), future AI models which also can consider RMSF result should be trained to design novel anti-viral drugs. In order to improve the performance of the ML models, larger and more diverse training datasets should be incorporated including experimental data and more simulation data, and more advanced AI architectures should be explored for designing hBD-2-based AMPs with enhanced binding affinity with RBD.

## Data Availability

The datasets presented in this study can be found in online repositories. The names of the repository/repositories and accession number(s) can be found in the article/Supplementary Material.
