# Supplementary material for: Human β defensin type 2 based novel antimicrobial peptide prediction using both molecular dynamics simulation and artificial intelligence methods
Source: Front Chem. 2026 Sep 29;14:1913090. doi: 10.3389/fchem.2026.1913090 (PMC13660420; doi:10.3389/fchem.2026.1913090)
Supplement: Supplementary file 1 [file DataSheet1.pdf]

Supplemental Materials

Human Beta Defensin Type 2 Based Novel Antimicrobial Peptide Prediction using Both Molecular Dynamics Simulation and Artificial Intelligence Methods

Ishrat Jahan[a], Hailemicael Lulseged Yimer[b], Kushal Badal[b], Michaela Brady[a], Jason J. Guo[c], Bryce Richardson[c], Letu Qingge[b], Liqun Zhang[a,c]

[a] Chemical Engineering Department, University of Rhode Island, Kingston, RI, USA, 02881

[b] Department of Computer Science, North Carolina Agricultural and Technical State University, Greensboro, NC, USA, 27411

[c] Barnett Institute for Chemical and Biological Analysis, Department of Chemistry and Chemical Biology, Northeastern University, 360 Huntington Avenue, Boston, MA, USA, 02115

[d] Chemical Engineering Department, Hampton University, Hampton, VA, USA, 23668

Tables

Table S1: Summary of hBD-2 block-mutant sequences (only RES18-RES30 region shown since the other section sequences are the same as those of hBD-2 wildtype) and the scores from AlphaFold2 prediction.

Mutants #	Sequences of Mutation (RES18-RES30)	pLDDT score(AF2)	pTM score	HADDOCK Score
Wildtype	VFCPRRYKQIGTC(original)	/	/	/
Seq1	IKKHWLLFWNYRK	52.9	0.256	-91.9±13.5
Seq2	VFSPRRYKQIGTS	94.9	0.702	-75.5±6.8
Seq3	AAAAAAAAAAAAA	66.7	0.424	-79.2±3.8
Seq4	VACARAYAQAGAC	95.8	0.721	-70.9±5.5
Seq5	IAKAWALAWAYAK	54.8	0.266	-82.4±3.5
Seq6	AKAHALAFANARA	62.9	0.355	-73.2±5.0
Seq7	VFAPRAYKAIGAC	95.3	0.717	-75.3±3.1
Seq8	AFCARRAKQAGTA	95.3	0.701	-76.5±2.0
Seq9	VACPARYAQIATC	95.7	0.703	-70.2±1.5
Seq10	IKAHWALFANYAK	59.7	0.284	-85.3±2.5
Seq11	AKKAWLAFWAYRA	58.5	0.284	-84.1±7.3
Seq12	IAKHALLAWNARK	65	0.35	-84.7±1.6
Seq13	IFCPWRYKWIGTK	87.3	0.59	-82.6 ± 4.5
Seq14	VFKPRRLKQIYTC	83.8	0.53	-105.3± 1.5
Seq15	VKCPRLYKQNGTC	96.2	0.725	-120.2± 4.3
Seq16	VFCHRRYFQIGRC	95.6	0.716	-116.1±9.9
Seq17	AKKHALLFANYRA	62.1	0.338	-128.5±8.3
Seq18	IAKHWALFWAYRK	59	0.261	-126.0±3.9
Seq19	IKAHWLAFWNARK	63.3	0.347	-130.7±4.0
Seq20	IKKAWLLAWNYAK	59	0.266	-124.9±8.6
Seq21	VFKPWHLHQIYRK	75.8	0.439	-117.3±3.7
Seq22	VFKPWLYFQIGTC	87.2	0.597	-96.2±5.5
Seq23	VFIPWLLFQIYRK	77.7	0.463	-101.6±9.5

Seq24	VPCPPAYKVIGTC	96.1	0.725	-87.5±6.8
Seq25	VPCPPCYHCIGTC	94.6	0.688	-108.5±4.9
Seq26	VFIPWRYHQIYRC	87	0.595	-107.8±12.4
Seq27	VFIPWHLHQIYRC	87.6	0.594	-91.1±1.9
Seq28	VFIPWLYFQIGRK	83.8	0.541	-112.4±6.0
Seq29	VFIPRLYFQIYTK	80.8	0.529	-95.9±10.0
Seq30	VFKPWHYKQIGRK	79.6	0.489	-122.3±4.1
Seq31	VFKPRLLKQIYTK	73.7	0.425	-109.3±8.9
Seq32	VFKPWRLKQIYRC	81.8	0.518	-100.9±8.3

Table S2: The average backbone RMSD, ΔG (kcal/mol), average number of hydrogen bonds, and buried surface area result of hBD-2 block-mutants interacting with RBD. The sequences of hBD-2 in the RES18-RES30 region are based on our manual prediction, which are also shown in Table S1. 7 sequences had rerun, and the rerun results are shown below the corresponding original run, which are highlighted in yellow. Among those 7 reruns, Seq26 showed stable binding in both the original and rerun simulations, which are in red.

Seq. No.	Block-mutations sequence (RES18-30)	RMSD (Å)	ΔG_{MD} (kcal/mol)	#hBonds	BSA (Å^2)
Wildtype	VFCPRRYKQIGTC (original)	2.2	-40.17±6.2	3.05	8229.5
Seq1	IKKHWLLFWNYRK	3.7	-25.31±3.8	1.07	660.43
Seq2	VFSPRRYKQIGTS	3.6	-28.57±9.6	3.07	433.15
Seq3	AAAAAAAAAAAAA	7.4	0	0.44	329.94
Seq4	VACARAYAQAGAC	4.3	-25.39±5.6	1.09	577.09
Seq5	IAKAWALAWAYAK	3.0;<br>4.8	-35.16±4.5;<br>-25.98±8.3	0.64;<br>1.17	662.89;<br>550.80
Seq6	AKAHALAFANARA	9.9	-5.69±6.5	1.17	577.09
Seq7	VFAPRAYKAIGAC	6.5	0.0	2.64	326.88
Seq8	AFCARRAKQAGTA	2.5;<br>8.1	-33.2±7.4;<br>-18.18±10.2	3.49;<br>1.05	615.16;<br>339.90
Seq9	VACPARYAQIATC	2.1	-30.77±6.2	2.70	624.39
Seq10	IKAHWALFANYAK	3.9;<br>4.0	-33.27±5.7;<br>-20.43±6.5	2.80;<br>0.97	613.11;<br>407.96
Seq11	AKKAWLAFWAYRA	6.5	0	1.15	519.63
Seq12	IAKHALLAWNARK	4.5	-7.81±7.2	0.91	254.29
Seq13	IFCPWRYKWIGTK	9.8	0	1.62	403.09
Seq14	VFKPRRLKQIYTC	2.6	-27.95±10.7	1.56	292.53
Seq15	VKCPRLYKQNGTC	11.8	0	1.64	381.67
Seq16	VFCHRRYFQIGRC	9.7	0	2.02	412.80
Seq17	AKKHALLFANYRA	10.7	0	1.08	554.58
Seq18	IAKHWALFWAYRK	9.4	-18.38±5.8	1.34	342.36
Seq19	IKAHWLAFWNARK	2.6	-24.25±8.44	1.23	568.91
Seq20	IKKAWLLAWNYAK	11.4	0	0.47	138.30
Seq21	VFKPWHLHQIYRK	6.0	-0.01	1.26	699.17
Seq22	VFKPWLYFQIGTC	3.1;<br>3.5	-32.46±5.6;<br>-28.27±6.6	1.91;<br>1.12	792.85;<br>587.61
Seq23	VFIPWLLFQIYRK	4.6	-23.82±6.9	0.95	482.60
Seq24	VPCPPAYKVIGTC	10.9	0	1.31	518.62
Seq25	VPCPPCYHCIGTC	3.7	-31.8±5.4	2.25	670.26

Seq26	VFIPWRYHQIYRC	3.6; 2.1	-39.33±11.7; -40.30±10.2	1.54; 1.53	673.78; 639.10
Seq27	VFIPWHLHQIYRC	5.6	0	0.99	413.27
Seq28	VFIPWLYFQIGRK	11.5	0	1.40	352.92
Seq29	VFIPRLYFQIYTK	4.1; 3.0	-33.69±7.1; -26.47±7.8	3.15; 1.03	628.85; 524.47
Seq30	VFKPWHYKQIGRK	3.0; 3.4	-41.43±5.2; -28.45±10.4	2.08; 1.18	551.92; 529.06
Seq31	VFKPRLLKQIYTK	7.0	0	0.55	524.91
Seq32	VFKPWRLKQIYRC	4.5	-9.97±8.2	2.16	453.28

Table S3. CNN Model Hyperparameters applied in the modelling.

Hyper Parameter	Value
Learning Rate	0.001
Number of Conv1D layers	2
Conv1D Layer 1 filters	32
Conv1D Layer 2 filters	64
Conv1D Kernel size	3
Dense Layer 1 neurons	64
Output Layer neurons	1
Activation Function (Conv1D, Dense)	ReLU
Output Activation Function	Linear
Optimizer	Adam
Loss Function	Mean Squared Error
Batch Size	32
Number of Epochs	100
Dataset Split	80% Training / 20% Test
Validation Split	20% of Training

Table S4. LSTM Model Hyperparameters applied in the modelling.

Hyper Parameter	Value
Learning Rate	0.001
Embedding Dimension	10
Number of LSTM Layers	2
LSTM Hidden Units	64
Dropout Rate	0.2
Regression Layer 1 neurons	32
Regression Layer 2 neurons	1
Activation Function (Regression Layers)	ReLU
Output Activation Function	Linear
Optimizer	Adam
Loss Function	Mean Squared Error
Batch Size	32
Number of Epochs	100
Dataset Split	80% Training / 20% Test

Table S5: Summary of 30 CNN model (top) and LSTM model (bottom) predicted hBD-2 mutant sequences for AlphaFold2 prediction and the scores from the AlphaFold2 prediction. The sequences having difference from the wildtype are shown, which are predicted based on Machine Learning and AI model.

Sequence ID	hBD2 multiple mutants Sequence (res18-30)	pLDDT Score	pTM Score	Confidence Ranking
Wildtype	VFCPRRYKQIGTC	/	/	/
ML-Seq1	LLGNQDPIHEFGF	59.6	0.303	1
ML-Seq2	MESTYPTYHGSFM	65.8	0.419	1
ML-Seq3	TQGSMPLMDKKTH	64.1	0.396	1
ML-Seq4	WHAGNAEKVEARH	79	0.509	1
ML-Seq5	FFHYGYRYQIAWL	79.8	0.496	1
ML-Seq6	RCGQWCWDGKADI	71.4	0.449	1
ML-Seq7	HMHNVAYEMMERE	63.8	0.363	1
ML-Seq8	MIMGKWCPGIRRQ	62.5	0.320	1
ML-Seq9	LVLQQVGITLDRK	56.8	0.292	1
ML-Seq10	DSQRPAEEDCIYW	71.7	0.436	1
ML-Seq11	QHEPACQKQEKKG	70.9	0.396	1
ML-Seq12	GQKPPRKSTFWMD	62.2	0.324	1
ML-Seq13	WEMDFYLQFEVCC	72.6	0.428	1
ML-Seq14	FWKFDGRKQQFII	64.2	0.379	1
ML-Seq15	LSCDKLPKPFSEW	69.9	0.406	1
ML-Seq16	ECATCECWLKCQS	67.4	0.376	1
ML-Seq17	KHGWFDCYDVDMY	72.9	0.421	1
ML-Seq18	TDLRFDGYLEVNS	76.6	0.428	1
ML-Seq19	EISNFDKYSEWWC	76.7	0.494	1
ML-Seq20	KEKTFFDYFHTTH	61.3	0.378	1
ML-Seq21	VWASGFCYHKETC	74.7	0.458	1
ML-Seq22	EREQEEDYKETCA	68.4	0.373	1
ML-Seq23	DIFYLLGYWDTKE	55.3	0.29	1
ML-Seq24	MNEVHDLYNIVYY	76.6	0.457	1
ML-Seq25	ALKKAELYSKCDF	63.1	0.338	1
ML-Seq26	KMLQFHNDGYAYI	57.3	0.309	1
ML-Seq27	SVAWHDHWPDQFY	58.3	0.322	1
ML-Seq28	AHQWIHKYYFLRY	61.7	0.301	1
ML-Seq29	LENSMFDYGVKML	64.4	0.344	1
ML-Seq30	TNRMIDCYGTCMT	80.2	0.525	1

Table S6: The average backbone RMSD of the ML predicted hBD-2 mutants bound with RBD complexes and for some repeat runs, which has the average RMSD of the hBD2-RBD complex smaller than 5.0 Å highlighted in red and RMSD higher than 10Å highlighted in blue, the repeat result highlighted in yellow; the ΔG (kcal/mol) of the complex predicted from ML and from the rerun MD using GMX/MM-PBSA method, along with average number of hydrogen bonds and buried surface area (BSA) calculations in the last 100 ns of simulations.

Label and Seq. of hBD-2 mutants	RMSD; RMSD of repeat (**Å**)	ΔG_{ML}; ΔG_{MD}; repeat (kcal/mol)	#hBonds	BSA ($Å^2$)
Wildtype	2.1; 3.6	N/A ; -40.17±6.2;	3.05	8229.5

{*VFCPRRYKQIGTC*}		-38.9±7.6		
ML-Seq1 {*LLGNQDPIHEFGF*}	5.8	-33.97; -11.57±13.3	1.52	271.95
ML-Seq2 {*MESTYPTYHGSFM*}	3.0; 2.0	-31.99; -31.63±6.8; -24.8±6.2	1.78	762.54
ML-Seq3 {***TQGSMPLMDKKTH***}	2.0; 3.7	-28.03; -41.56±8.3; -39.8±10.8	4.54	633.44
ML-Seq4 {*WHAGNAEKVEARH*}	4.0	-27.18; -25.45±4.6	0.84	620.11
ML-Seq5 {***FFHYGYRYQIAWL***}	1.6; 2.6	-24.36; -30.26±7.6; -31.3±6.4	2.81	621.21
ML-Seq6 {*RCGQWCWDGKADI*}	6.7	-23.95; -5.08±8.1	3.17	191.92
ML-Seq7 {*HMHNVAYEMMERE*}	3.4; 7.2	-20.63; -55.39±16.4; -24.8±8.1	2.58	785.08
ML-Seq8 {***MIMGKWCPGIRRQ***}	2.4; 5.3	-19.79; -34.97±6.0; -42.4±7.5	3.46	582.33
ML-Seq9 {*LVLQQVGITLDRK*}	11.6	-16.02; -25.07±18.0	3.40	635.68
ML-Seq10 {*DSQRPAEEDCIYW*}	2.7	-14.86; -21.34±10.4	2.05	477.54
ML-Seq11 {*QHEPACQKQEKKG*}	5.8	-14.76; -27.49±7.1	0.78	645.99
ML-Seq12 {*GQKPPRKSTFWMD*}	9.3	-12.64; -16.31±4.8	1.52	348.50
ML-Seq13 {*WEMDFYLQFEVCC*}	5.5; 4.3	-8.64; -40.75±6.2; -28.8±7.1	0.66	791.47
ML-Seq14 {*FWKFDGRKQQFII*}	11.3	-7.51; -20.1±6.5	1.22	486.69
ML-Seq15 {*LSCDKLPKPFSEW*}	3.2	-6.02; -18.9±9.2	2.46	468.90
ML-Seq16 {*ECATCECWLKCQS*}	6.3	-84.79; -21.27±5.8	3.41	397.68
ML-Seq17 {*KHGWFDCYDVDMY*}	3.9	-73.84; -29.12±10.7	2.42	687.56
ML-Seq18 {*TDLRFDGYLEVNS*}	3.3	-72.58; -13.08±6.4	1.64	453.55
ML-Seq19 {*EISNFDKYSEWWC*}	4.0	-72.16; -22.05±5.9	1.71	588.58
ML-Seq20 {*KEKTFFDYFHTTH*}	9.9; 6.3	-72.08; -52.01±6.3; -14.5±8.2	0.90	726.84
ML-Seq21 {*VWASGFCYHKETC*}	3.0	-68.30; -28.29±5.7	1.62	644.47

ML-Seq22 {*EREQEEDYKETCA*}	4.3	-67.85; -19.65±9.6	1.21	530.61
ML-Seq23 {*DIFYLLGYWDTKE*}	10.5	-67.34; -15.41±6.5	0.85	383.15
ML-Seq24 {*MNEVHDLYNIVYY*}	2.4; 3.3	-67.25; -41.13±7.4; -26.1±6.8	4.90	664.82
ML-Seq25 {*ALKKAELYSKCDF*}	3.2	-67.15; -29.0±7.0	0.57	702.11
ML-Seq26 {*KMLQFHNDGYAYI*}	3.3	-65.38; -26.72±6.1	1.05	767.28
ML-Seq27 {*SVAWHDHWPDQFY*}	8.1	-64.41; -9.29±7.3	1.05	371.42
ML-Seq28 {*AHQWIHKYYFLRY*}	11.1	-63.45; -23.7±8.9	1.36	507.55
ML-Seq29 {*LENSMFDYGVKML*}	7.8	-62.08; -8.19±3.4	1.41	49.26
ML-Seq30 {*TNRMIDCYGTCMT*}	4.4	-61.77; -21.1±8.1	1.80	464.32

Table S7: Summary of 20 LSTM model predicted hBD-2 mutant sequences for AlphaFold2 prediction and the scores from the AlphaFold2 prediction. The sequences having difference from the wildtype are shown, which are predicted based on LSTM model.

Sequence ID	hBD2 multiple mutants Sequence (res18-30)	pLDDT Score	pTM Score	Confidence Ranking
Wildtype	VFCPRRYKQIGTC	/	/	/
LS-Seq1	DRTPIKFVASMAE	82.9	0.52	1
LS-Seq2	LGWMDECYHYGNV	88.7	0.61	1
LS-Seq3	QTNWFPQVGITWE	78.0	0.49	1
LS-Seq4	TSVPKVQSYEMII	77.1	0.48	1
LS-Seq5	FIQRDIYTCHASI	82.2	0.54	1
LS-Seq6	FYWWMYANAMSKH	66.9	0.357	1
LS-Seq7	NQSWGEIHNFGMC	85.4	0.592	1
LS-Seq8	HPVINSTMQAKWC	76	0.484	1
LS-Seq9	FQMNQGYYAKPYD	85.6	0.57	1
LS-Seq10	NTSELQNYEFIDE	83.9	0.536	1
LS-Seq11	ANWIFGRFLNNSE	58.9	0.287	1
LS-Seq12	CPVWTNAMAFCMT	83.8	0.542	1
LS-Seq13	LRIMEEMPYIHYV	79.2	0.533	1
LS-Seq14	VPFPCDEGADNKD	71.2	0.455	1
LS-Seq15	EYKVIVILRAVTM	60.3	0.301	1
LS-Seq16	KTQTPDEWFIGHV	91.7	0.656	1
LS-Seq17	QTNCHDRKIGSQL	85.8	0.602	1
LS-Seq18	WACQHGRPAKVAY	74.4	0.45	1
LS-Seq19	LYHLCWMNKIVFK	80.4	0.495	1

LS-Seq20	APTNHAKFMKRTG	67.3	0.365	1

Table S8. LSTM model predicted sequences initiated simulation result, including the binding interaction energy, RMSD and the number of hydrogen bonds formed between hBD-2 mutant and RBD, and the buried surface area (BSA) of the complex for LS-seq1 to LS-Seq20 based on 500 ns of simulations with the initial sequences of hBD-2 mutant from the LSTM model prediction. The results from LS-Seq11, LS-Seq14 and LS-Seq20 are in yellow with both the original and rerun results shown.

Sequence and labeling	ΔG(kcal/mol)	RMSD (Å)	#hBonds	BSA (Å^2)
LS-Seq1: DRTPIKFVASMAE	-28.65±6.6	4.8	1.28±0.95	507±62.1
LS-Seq2: LGWMDECYHYGNV	-20.51±10.1	6.9	0.85±0.90	437.14±173.1
LS-Seq3: QTNWFPQVGITWE	-27.23±11.2	6.1	0.87±1.11	570.0±126.0
LS-Seq4: TSVPKVQSYEMII	-17.63±6.1	6.0	0. 51±0.67	449.8±109.8
LS-Seq5: FIQRDIYTCHASI	-24.35±5.8	4.0	0.70±0.80	581.21±102.1
LS-Seq6: FYWWMYANAMSKH	-23.09±13.4	8.5	1.20±1.28	425.37±209.2
LS-Seq7: NQSWGEIHNFGMC	-26.42±9.2	8.9	1.17±1.11	481.05±114.3
LS-Seq8: HPVINSTMQAKWC	-23.94±7.1	5.8	0.64±0.77	562.87±89.4
LS-Seq9: FQMNQGYYAKPYD	-27.13±13.3	4.4	1.10±1.06	574.78±192.3
LS-Seq10: NTSELQNYEFIDE	-26.69±7.0	4.1	0.82±0.86	631.38±91.31
LS-Seq11: ANWIFGRFLNNSE	-43.04±9.8; -29.63±11.3	5.5; 5.2	1.57±1.22; 1.18±1.15	704.09±93.11; 575.08±192.74
LS-Seq12: CPVWTNAMAFCMT	-33.13±10.6	6.7	0.97±0.91;	598.22±121.1
LS-Seq13: LRIMEEMPYIHYV	-27.93±6.6	7.2	0.88±0.84	515.13±87.9
LS-Seq14: VPFPCDEGADNKD	-42.62±9.8; -27.11±8.4	4.7; 4.8	1.93±1.36; 0.71±0.81	776.23±114.04; 543.40±95.19
LS-Seq15: EYKVIVILRAVTM	-27.95±8.7	5.2	0.91±0.93	569.24±101.1
LS-Seq16: KTQTPDEWFIGHV	-19.38±9.7	6.1	0. 50±0.64	412.14±174.8
LS-Seq17: QTNCHDRKIGSQL	0.0	Dissociation	0.0	0.0
LS-Seq18: WACQHGRPAKVAY	-32.73±6.6	3.4	1.11±0.99	746.31±74.5
LS-Seq19: LYHLCWMNKIVFK	-35.02±7.5	3.5	1.41±1.14	800.77±86.3
LS-Seq20: APTNHAKFMKRTG	-42.94±7.8; -47.54±14.0	2.5; 6.1;	2.17±1.38; 1.78±1.21	601.14±70.09; 752.99±163.69

Table S9: MM-PBSA binding free energies decomposition for the WT and block-mutants (along with standard error) that are showing significant negative contribution. The total binding free energy (ΔG_{total}) is resolved into electrostatic (ΔE_{ele}), van der Waals (ΔE_{vdw}), polar solvation (ΔE_{polar}), and non-polar solvation ($\Delta E_{non\text{-}polar}$) contributions to identify the dominant interactions governing complex stability. All values are expressed in kcal/mol.

Sequences	ΔE_{ele}	ΔE_{vdw}	ΔE_{polar}	$\Delta E_{non\text{-}polar}$	ΔG_{total}
**WT**	-51.49 ± 30.48	-59.31 ± 6.00	80.45 ± 28.29	-9.81 ± 0.84	-40.17 ± 6.17

**Seq1**	141.44 ± 42.44	-58.84 ± 5.21	-99.95 ± 41.34	-7.96 ± 0.63	-25.31 ± 3.79
**Seq5**	52.20 ± 22.97	-63.74 ± 4.64	-15.99 ± 21.70	-7.63 ± 0.50	-35.16 ± 4.52
**Seq8**	-13.04 ± 46.22	-45.49 ± 6.69	32.43 ± 42.36	-7.11 ± 0.98	-33.20 ± 7.36
**Seq10**	-10.36 ± 26.75	-59.73 ± 5.26	45.27 ± 24.42	-8.45 ± 0.59	-33.27 ± 5.70
**Seq22**	-27.76 ± 20.23	-56.82 ± 6.77	60.60 ± 19.57	-8.48 ± 1.04	-32.46 ± 5.59
**Seq26**	-1.61 ± 40.64	-75.85 ± 15.60	48.12 ± 40.37	-9.98 ± 2.27	-39.33 ± 11.70
**Seq29**	7.41 ± 41.36	-57.22 ± 8.42	24.44 ± 36.79	-8.32 ± 1.10	-33.69 ± 7.07
**Seq30**	18.47 ± 28.33	-54.65 ± 4.86	2.25 ± 27.47	-7.50 ± 0.59	-41.43 ± 5.21

Table S10: MM-PBSA binding free energies decomposition for the WT and ML predicted mutants (along with standard error) that are showing significant negative contribution. The total binding free energy (ΔG_{total}) is resolved into electrostatic (ΔE_{ele}), van der Waals (ΔE_{vdw}), polar solvation (ΔE_{polar}), and non-polar solvation ($\Delta E_{non\text{-}polar}$) contributions to identify the dominant interactions governing complex stability. All values are expressed in kcal/mol.

**Sequences**	**ΔE_{ele}**	**ΔE_{vdw}**	**ΔE_{polar}**	**$\Delta E_{non\text{-}polar}$**	**ΔG_{total}**
**WT**	-51.49 ± 30.48	-59.31 ± 6.00	80.45 ± 28.29	-9.81 ± 0.84	-40.17 ± 6.17
**ML-Seq2**	12.26 ± 25.94	-61.98 ± 7.40	26.63 ± 25.28	-8.53 ± 1.08	-31.63 ± 6.83
**ML-Seq3**	-119.60 ± 43.20	-45.66 ± 7.23	130.93 ± 41.92	-7.22 ± 0.96	-41.56 ± 8.32
**ML-Seq5**	-8.17 ± 28.06	-52.42 ± 7.95	37.27 ± 26.37	-6.94 ± 1.04	-30.26 ± 7.58
**ML-Seq7**	-116.28 ± 43.00	-69.09 ± 7.77	139.75 ± 32.54	-9.77 ± 1.12	-55.39 ± 16.38
**ML-Seq8**	-83.65 ± 40.04	-44.95 ± 9.17	100.17 ± 38.69	-6.54 ± 1.16	-34.97 ± 5.99
**ML-Seq13**	-85.93 ± 19.76	-66.38 ± 6.39	120.87 ± 18.69	-9.31 ± 0.72	-40.75 ± 6.19
**ML-Seq20**	-142.42 ± 29.87	-66.46 ± 5.44	166.30 ± 27.87	-9.43 ± 0.73	-52.01 ± 6.25
**ML-Seq24**	-112.28 ± 27.94	-52.34 ± 7.45	132.00 ± 25.22	-8.51 ± 0.93	-41.13 ± 7.37

Figures

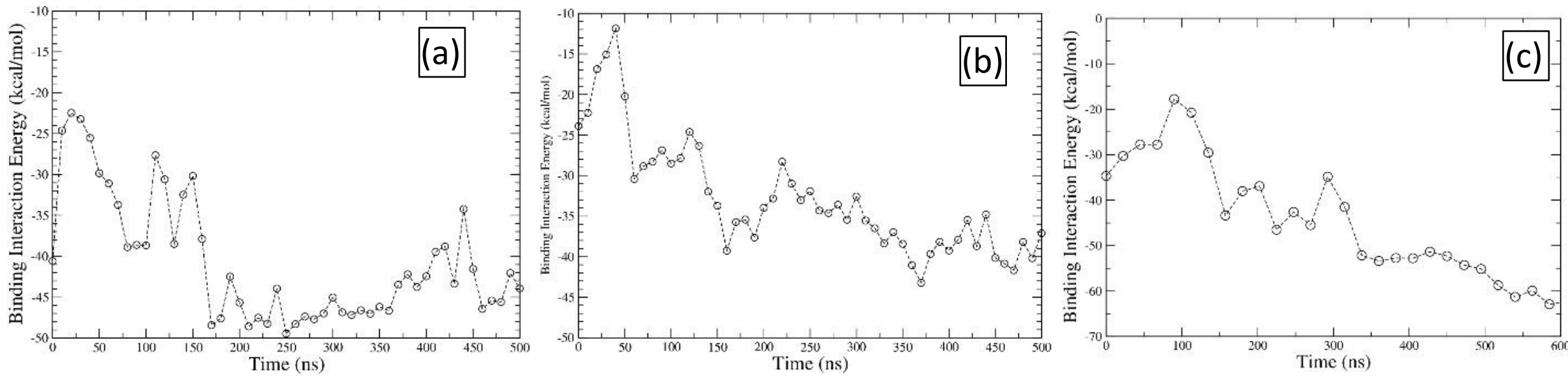

Figure S1. The block average of binding interaction energy for block-mutant Seq26(a), ML-Seq8(b) and LS-Seq20((c) during NAMD simulations.

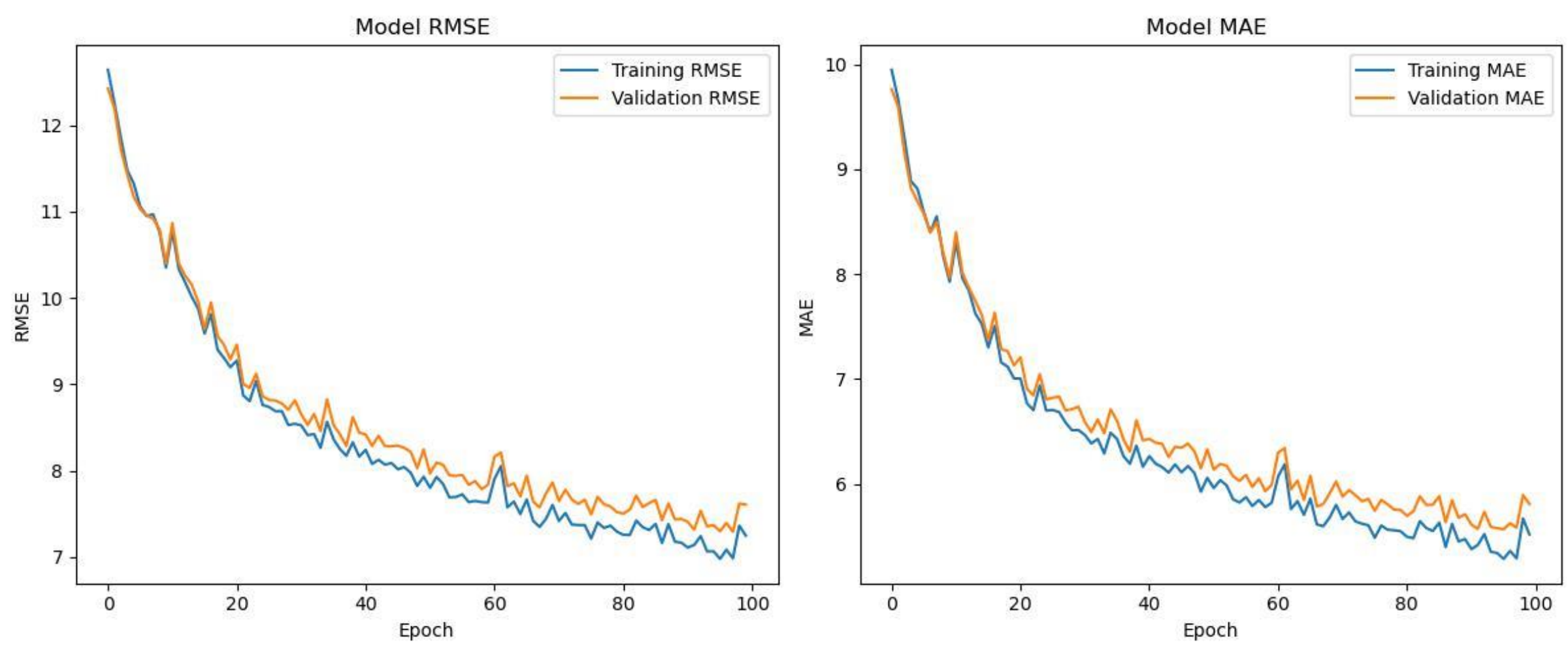

Figure S2. Training and validation metrics over 100 epochs: RMSE and MAE.

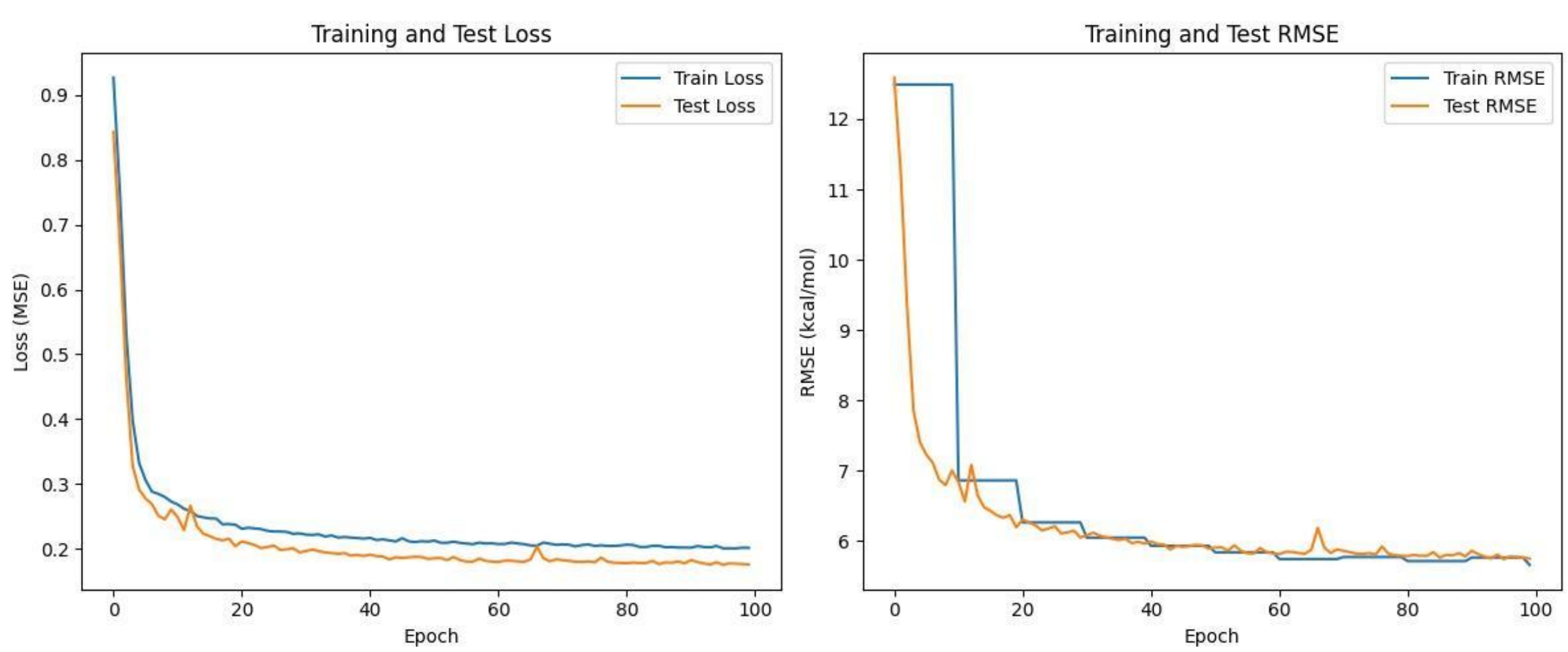

Figure S3: Training and validation metrics over 100 epochs: RMSE and MAE.

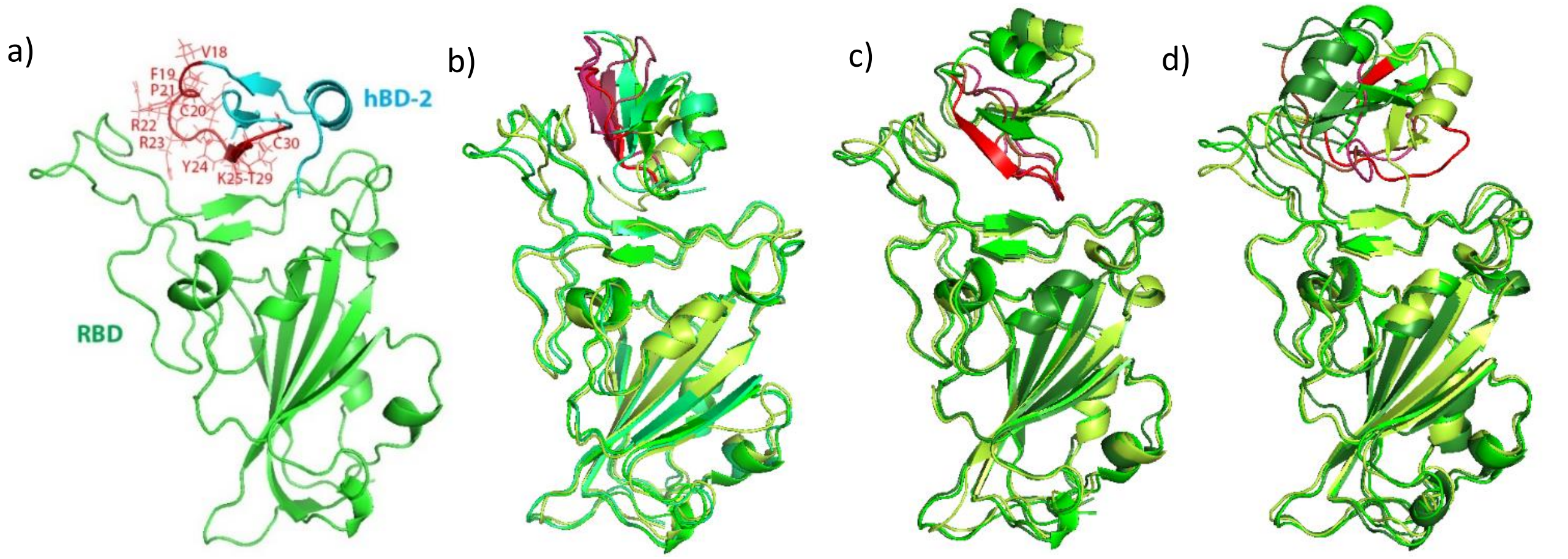

Figure S4. Binding structure of hBD-2 (cyan) with RBD (green) used as a starting structure for the MD simulations in wildtype (WT) and for spot-mutants with the binding region of V18-C30 highlighted in red lines and mostly labelled(a); Comparison of the binding structures of RBD bound with hBD-2 ML-Seq3 (b), ML-Seq5 (c), and ML-Seq8(d) predicted from ML in original structure (green), the last structure after 500 ns Gromacs simulation (dark green) and the last structure after the repeat NAMD simulation (limo) with the mutated sequences highlighted in red (initial structure), brown (original Gromacs run) and pink (repeat NAMD run).

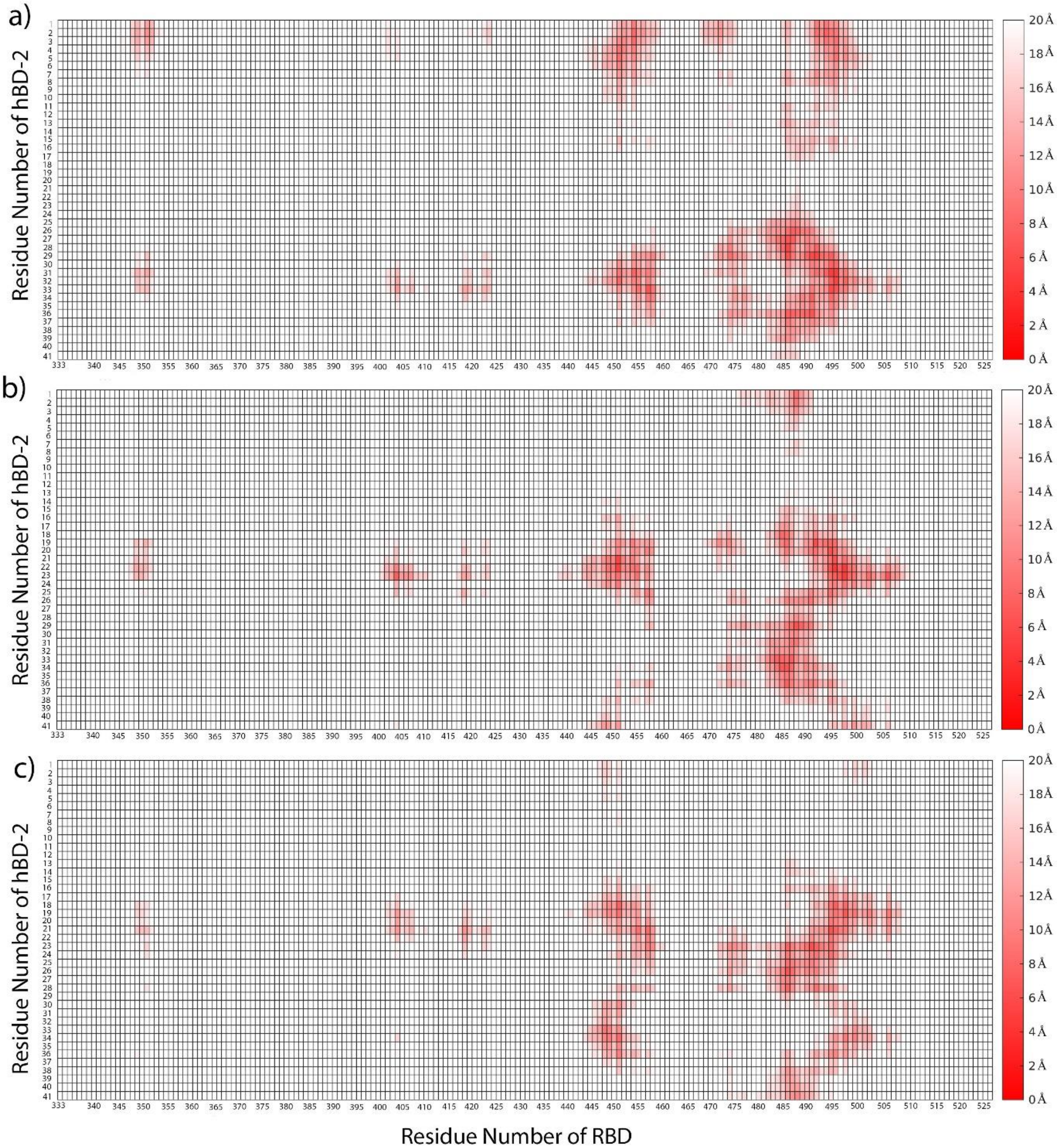

Figure S5. Comparison of distance maps of hBD-2 ML-seq3 (a), ML-seq5 (b) and ML-seq8 (c) bound with RBD based on rerun simulation results.

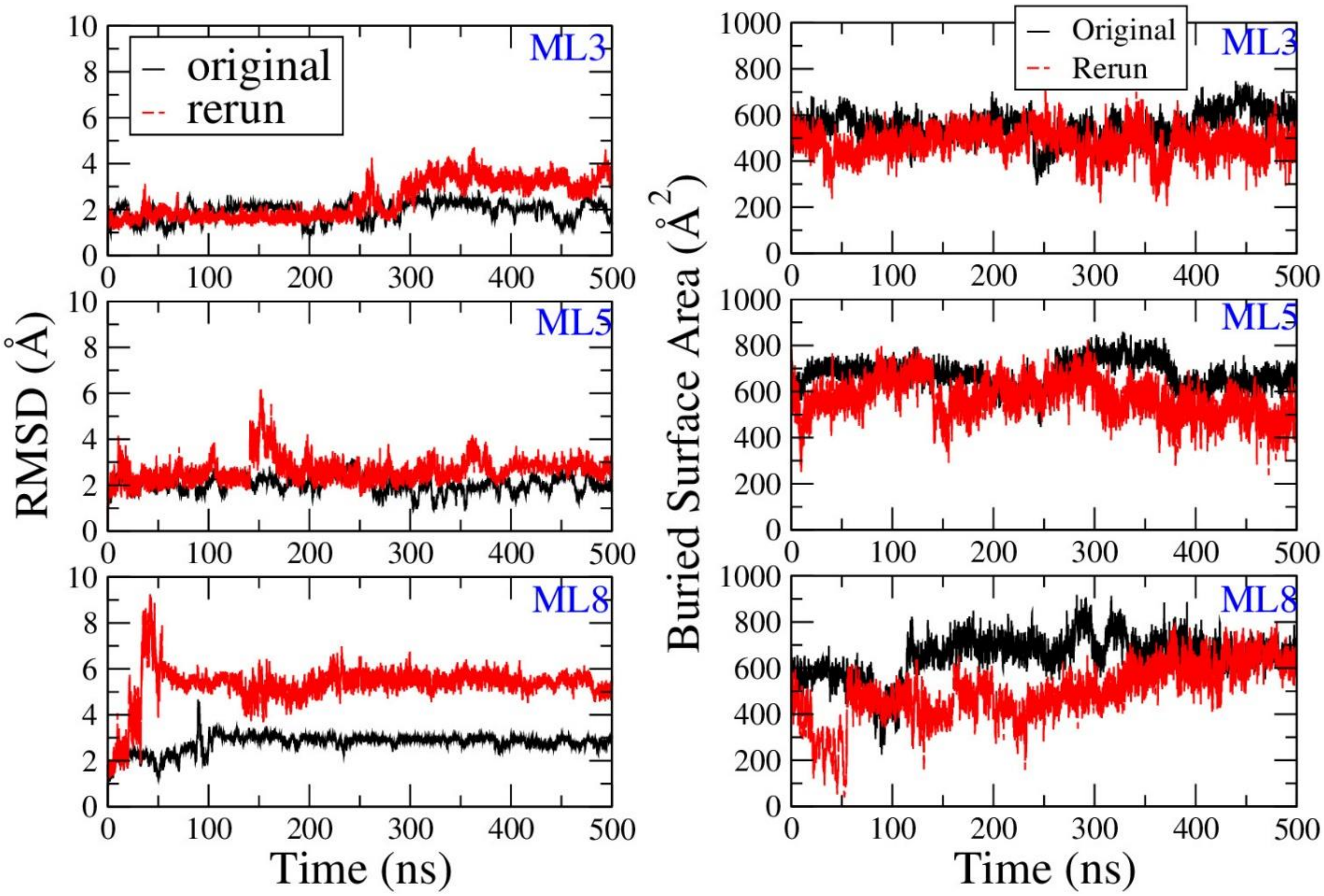

Figure S6. The RMSD (Left) of the complex and buried surface area (Right) of RBD bound with hBD-2 ML-Seq3, ML-Seq5, ML-Seq8 in the original and rerun simulations.

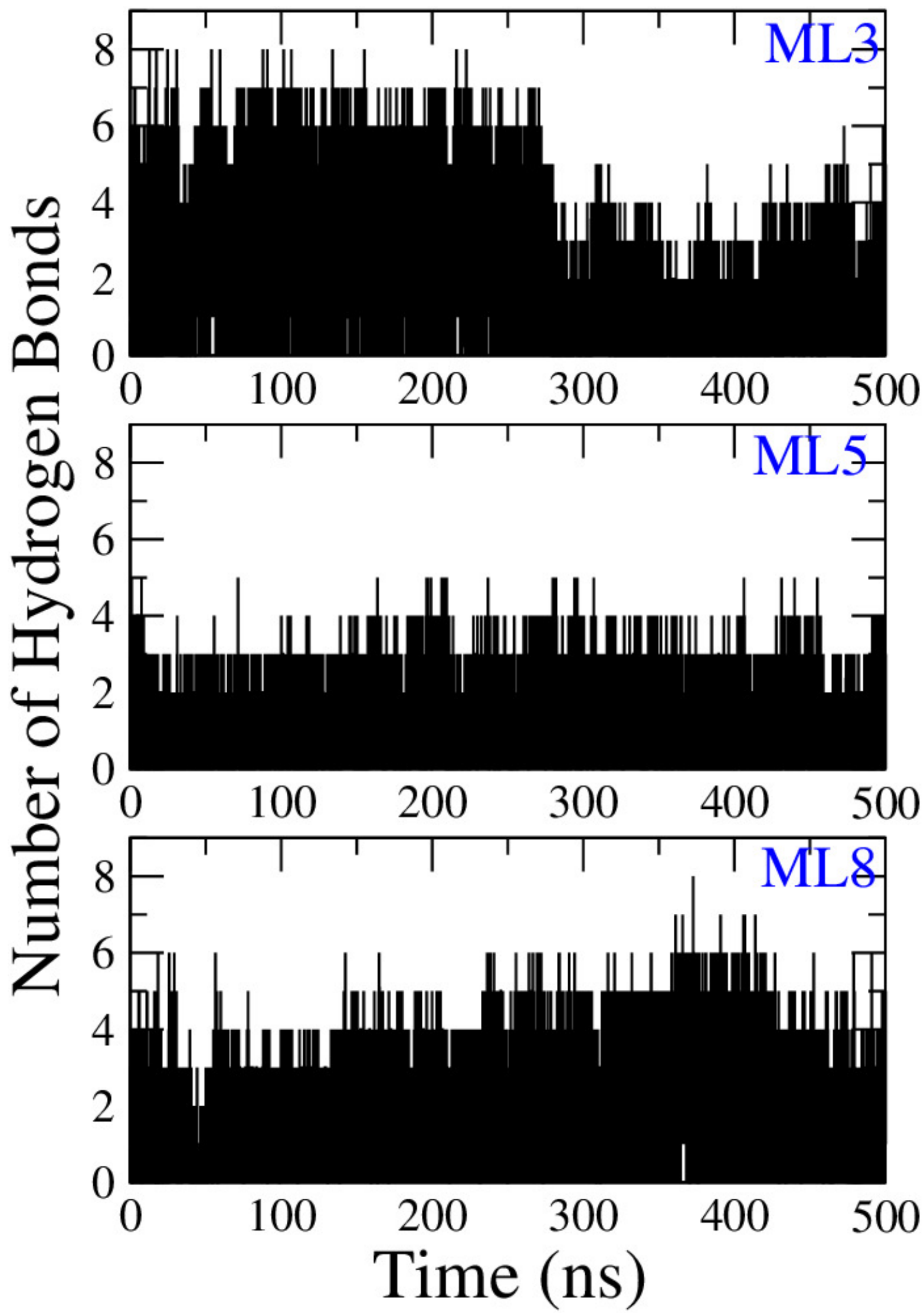

Figure S7. The number of hydrogen bonds formed between RBD and hBD-2 mutant ML-Seq3, ML-Seq5, and ML-Seq8 in rerun simulations.

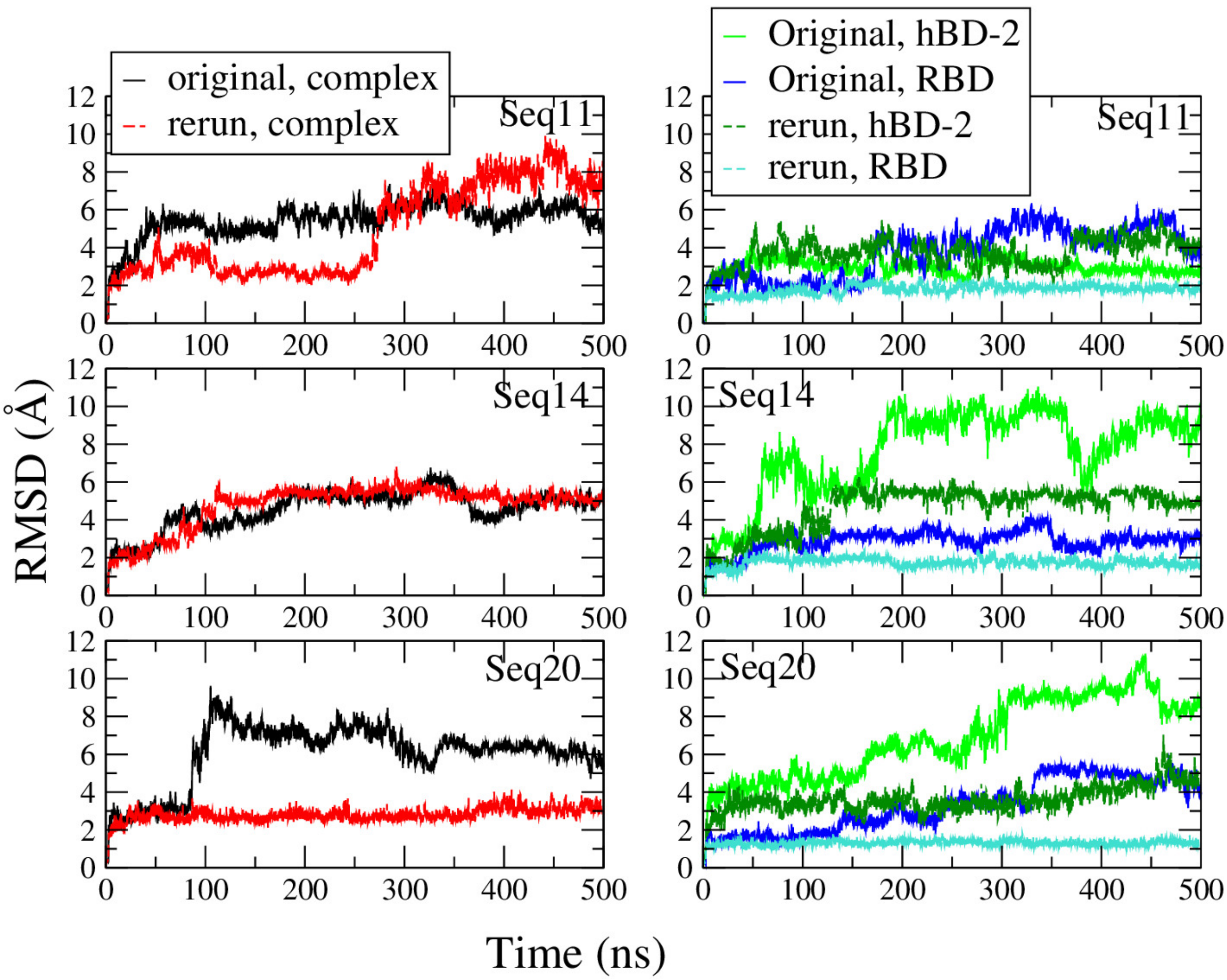

Figure S8. The RMSD of the complex of hBD-2 mutant and RBD, hBD-2 mutant and RBD in original run and rerun for LS-Seq11, LS-Seq14 and LS-Seq20.

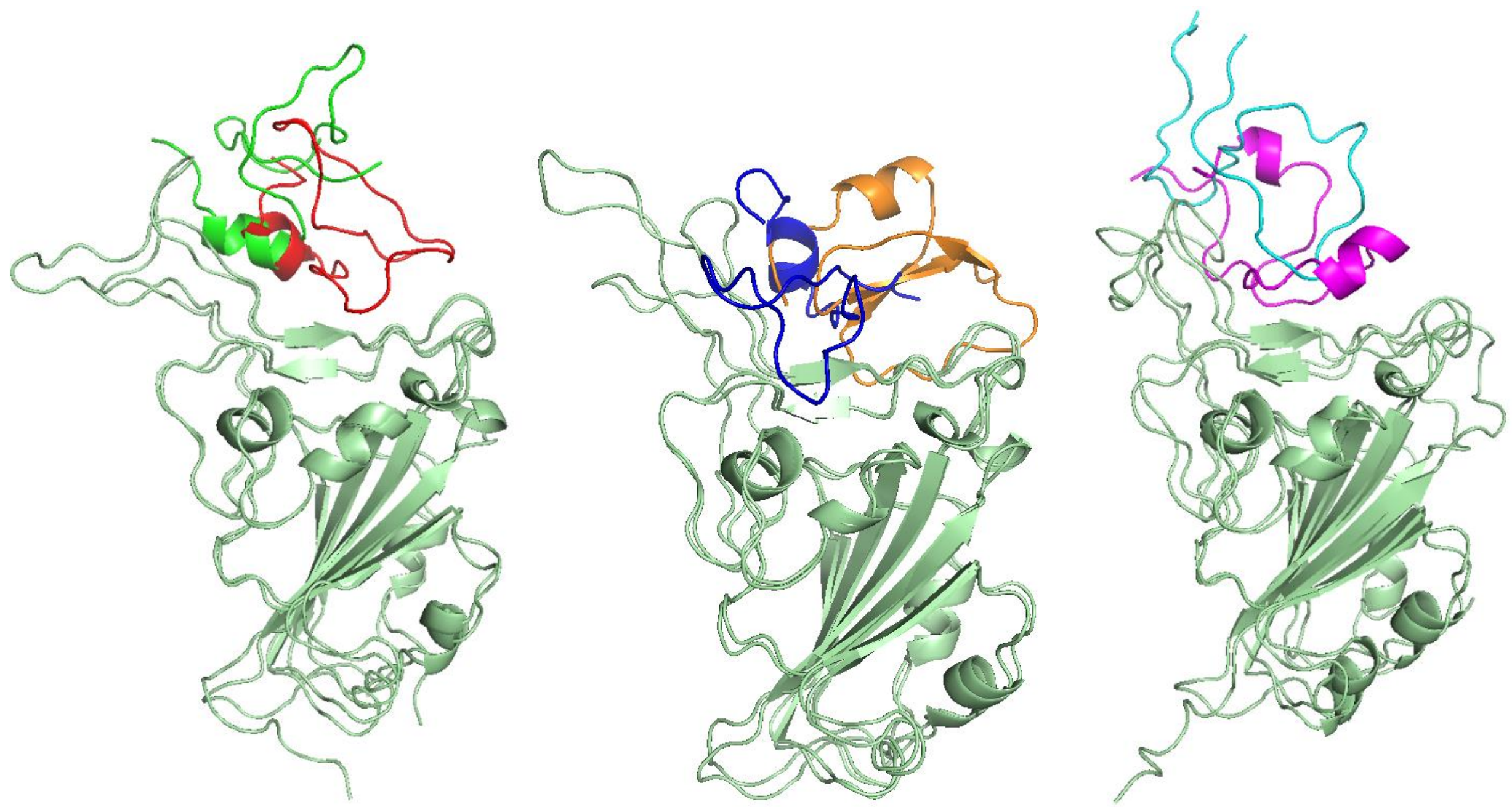

Figure S9. The comparison of the last complex structures output for LS-Seq11(Left), LS-Seq14(middle), and LS-Seq20 (Right) systems based on the original and rerun simulations. All the RBD are aligned and are shown in light green, while hBD-2 mutants from different simulations are shown in different colors. The hBD-2 LS-Seq11 from the original simulation is shown in red, from rerun in green, the LS-Seq14 from original prediction in blue, from the rerun in orange, the LS-Seq20 from original run prediction in magenta, from the rerun in cyan.

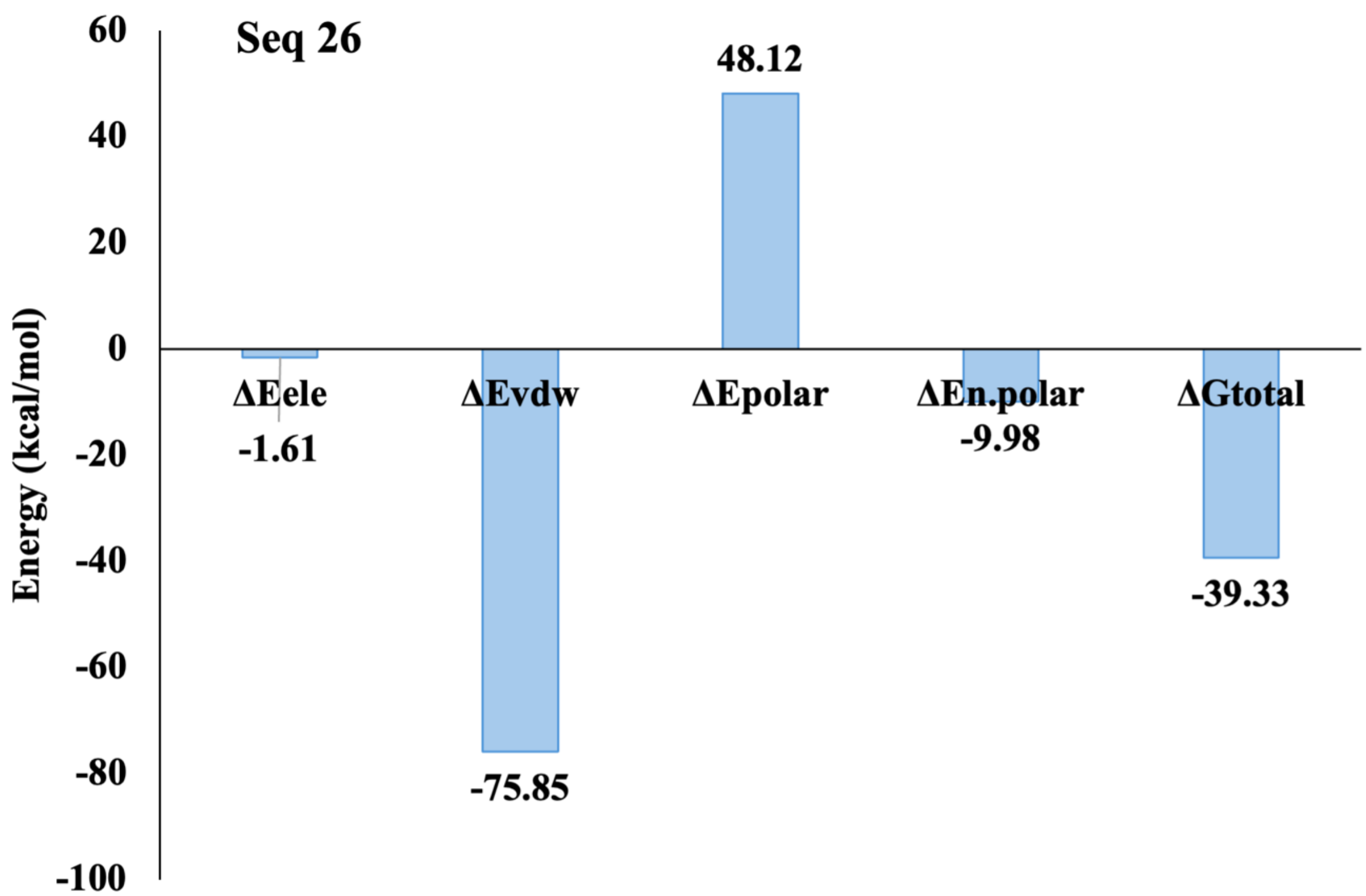

Figure S10: MM-PBSA binding free energy decomposition for Seq26. Favorable contributions arise mainly from van der Waals (ΔE_{vdw}) and non-polar solvation ($\Delta E_{non\text{-}polar}$) energies, whereas polar solvation (ΔE_{polar}) opposes binding. The minimal electrostatic contribution (ΔE_{ele}) indicates that hydrophobic/van der Waals interactions are the principal drivers of complex stability.

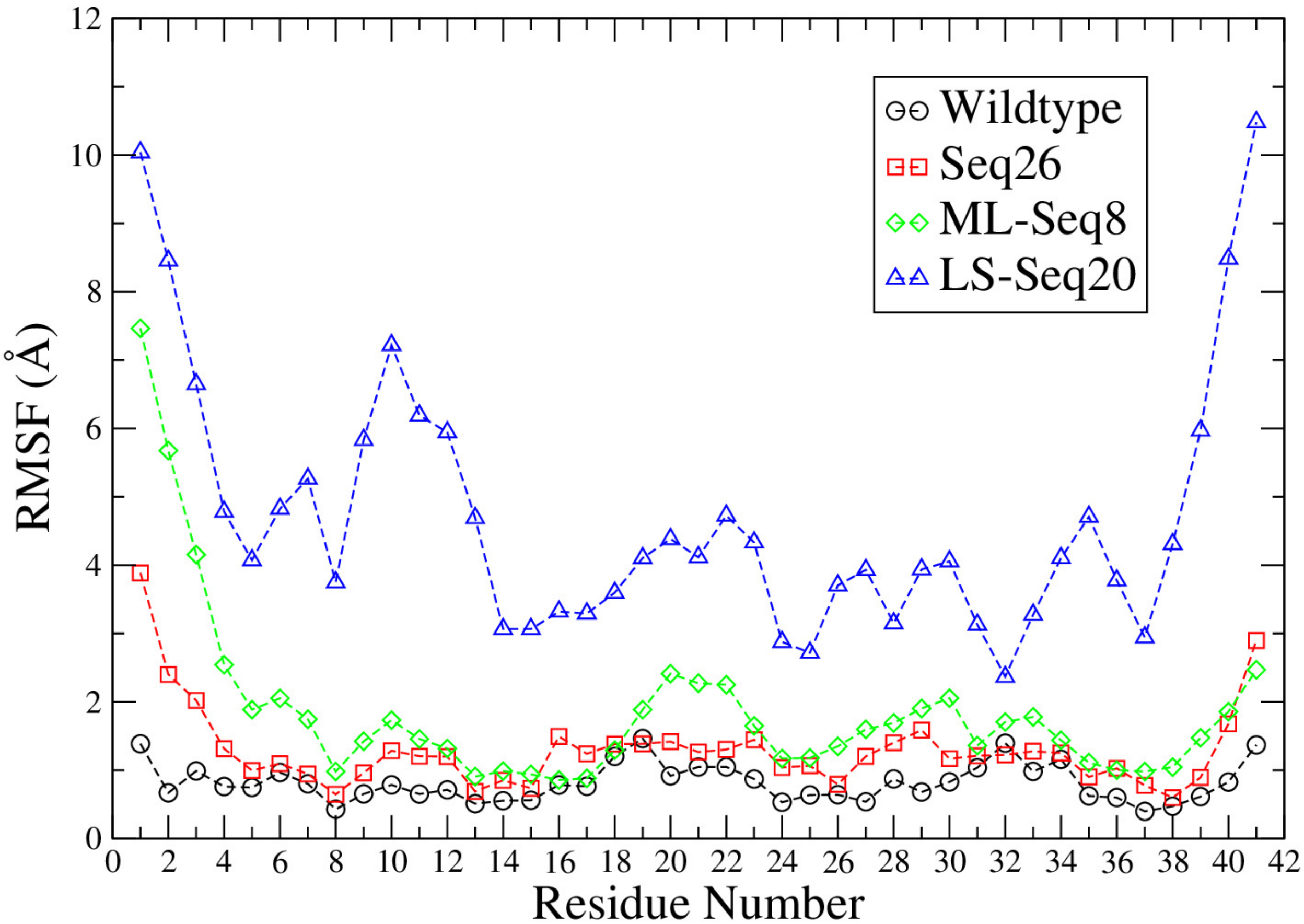

Figure S11. RMSF comparison between Seq26, ML-Seq8, LS-Seq20 and hBD-2 wildtype bound with RBD based on rerun simulations.
